# Fine-tuning of AMPK–ULK1–mTORC1 regulatory triangle is crucial for autophagy oscillation

**DOI:** 10.1038/s41598-020-75030-8

**Published:** 2020-10-20

**Authors:** Marianna Holczer, Bence Hajdú, Tamás Lőrincz, András Szarka, Gábor Bánhegyi, Orsolya Kapuy

**Affiliations:** 1 Department of Medical Chemistry, Molecular Biology and Pathobiochemistry, Semmelweis University, Tűzoltó utca 37-47, Budapest, 1094 Hungary; 2 Laboratory of Biochemistry and Molecular Biology, Department of Applied Biotechnology and Food Science, Budapest University of Technology and Economics, Budapest, Hungary; 3 Pathobiochemistry Research Group of the Hungarian Academy of Sciences and Semmelweis University, Budapest, Hungary

**Keywords:** Cell biology, Systems biology

## Abstract

Autophagy is an intracellular digestive process, which has a crucial role in maintaining cellular homeostasis by self-eating the unnecessary and/or damaged components of the cell at various stress events. ULK1, one of the key elements of autophagy activator complex, together with the two sensors of nutrient and energy conditions, called mTORC1 and AMPK kinases, guarantee the precise function of cell response mechanism. We claim that the feedback loops of AMPK–mTORC1–ULK1 regulatory triangle determine an accurate dynamical characteristic of autophagic process upon cellular stress. By using both molecular and theoretical biological techniques, here we reveal that a delayed negative feedback loop between active AMPK and ULK1 is essential to manage a proper cellular answer after prolonged starvation or rapamycin addition. AMPK kinase quickly gets induced followed by AMPK-P-dependent ULK1 activation, whereas active ULK1 has a rapid negative effect on AMPK-P resulting in a delayed inhibition of ULK1. The AMPK-P → ULK1 ˧ AMPK-P negative feedback loop results in a periodic repeat of their activation and inactivation and an oscillatory activation of autophagy, as well. We demonstrate that the periodic induction of self-cannibalism is necessary for the proper dynamical behaviour of the control network when mTORC1 is inhibited with respect to various stress events. By computational simulations we also suggest various scenario to introduce “delay” on AMPK-P-dependent ULK1 activation (i.e. extra regulatory element in the wiring diagram or multi-phosphorylation of ULK1).

## Introduction

(Macro)autophagy, also called self-cannibalism, is an evolutionarily conserved cellular digestive process. During autophagy double membrane vesicles (i.e. autophagosomes) are formed around the unnecessary and/or damaged cellular components (such as various proteins or mitochondria)^1–3^. To ensure cellular homeostasis these elements are later delivered to and degraded by the lysosomes. Although cells have some basic autophagy even under physiological conditions, autophagy gets significantly activated by various stress events (i.e. starvation and growth factor deprivation)^3–5^. The intensity of autophagy is tightly regulated by the opposing effect of mTORC1 and AMPK kinases^3–5^. While AMPK enhances autophagy, mTORC1 keeps the self-cannibalism inactive at physiological conditions.

It is well-known that mTORC1 has a key role in controlling cell growth and cellular metabolism by integrating different external and internal signals, such as growth factors, amino acids, glucose and energy status^3–5^. mTOR is a serine/threonine protein kinase and the catalytic subunit of mTORC1 complex. To guaranty the precise regulation of mTOR pathway this complex contains various regulatory subunits (such as Raptor, MLST8, PRAS40, DEPTOR)^6^. At physiological conditions mTORC1 is kept active, meanwhile inactivation of mTORC1 leads to a general inhibition of protein translation in the cell, autophagy-dependent self-digestive process becomes fully active. Deprivation of mTORC1 pathway quickly down-regulates protein synthesis by de-phosphorylating the ribosomal protein S6 kinase (p70S6K) and the translation initiation factor 4E binding protein-1 (4E-BP1)^7,8^.

AMPK is a heterotrimeric protein complex and it has an essential role in maintaining energy homeostasis by sensing the change of cellular AMP/ATP ratio. When cellular AMP level is high (i.e. cellular energy level is low), the free AMP directly binds to AMPK and turns it on^9^. AMPK tightly controls ATP-consuming mechanisms, such as glycogen or protein syntheses; fatty acids and cholesterol syntheses due to Ser/Thr phosphorylation of key regulatory enzymes and therefore up-regulates processes that increase ATP level (i.e. glycolysis, β-oxidation) in the cell^10,11^.

Both mTORC1 and AMPK control autophagy via one of the key inducers of the complex required to autophagosome formation, called Unc-51 like autophagy activating kinase (ULK1/2)^12^. AMPK is able to promote the self-eating mechanism by phosphorylating ULK1^13–15^, while mTORC1 down-regulates the self-eating process under nutrient rich condition by phosphorylating ULK1^12,14,16,17^. However, the active ULK1 can inhibit both AMPK^18,19^ and mTORC1^20,21^. Besides, AMPK directly inhibits mTOR complex 1 via phosphorylation upon nutrient depletion^15,22^. Recently it has been shown both experimentally and theoretically that not only AMPK inhibits mTORC1, but mTORC1 has also a negative effect on AMPK resulting in a double negative feedback loop in the control network^23,24^. Ling et al. has revealed that mTORC1 down-regulates AMPK via direct phosphorylation on AMPK α subunit at Ser-345 which negatively affects cellular phosphorylation of AMPK catalytic subunit at Thr-172^24^. Upon glucose starvation AMPK inhibits mTORC1 either via the TSC1–TSC2 (hamartin-tuberin) complex or by direct phosphorylation of Raptor^14,22,25–27^. This inhibition is required for the dephosphorylation of ULK1 at Ser-757^14^, Ser-638 and Ser-758^28^ residues and it leads to an intensive development of the connection between ULK1 and AMPK^14,28^. Then AMPK phosphorylates ULK1 on different phosphorylation sites (e.g. Ser-555, Ser-777, Ser-317, Ser-467) and therefore promotes autophagy^14,25^.

Rapamycin treatment down-regulates the activity of mTORC1^29^ resulting in the de-phosphorylation of ULK1 at Ser-637, Ser-757^30^, corresponding to Ser-638 and Ser-758 residues on ULK1 in human cells^28^. Our previous studies have already shown that AMPK becomes active due to its phosphorylation at Thr-172 during rapamycin treatment. AMPK-P is able to phosphorylate ULK1 at Ser-555 followed by an intensive autophagic process^23,28^. Parallel with our results, in other studies an increased amount of the phosphorylated AMPK was observed upon rapamycin treatment^31^. However, another experimental data suggested that ULK1 and autophagy activation occurred in an AMPK independent manner^14^ and AMPK activation was not detected at all^32,33^.

Interestingly, a periodic activation of mTORC1 was observed during T cell proliferation. The early down-regulation and subsequent up-regulation of mTOR pathway seemed to be crucial both to enhance and maintain Treg cell proliferation^34^. It has been also shown that the intensity of autophagy varies throughout the day in several tissues suggesting its connection to the circadian clock. ULK1 is a target of C/EBPβ protein which has an essential role in controlling rhythmic expression of autophagy genes^35^. Recently, Nazio et al. has revealed that ULK1 level gets marked for proteasome-dependent degradation by the E3 ubiquitin ligase NEDD4L during EBSS treatment, meanwhile the transcription of ULK1 remains active. However the synthesized ULK1 is inhibited by mTORC1-dependent phosphorylation resulting in an oscillatory induction of autophagy during prolonged stress^36,37^.

Recently we have introduced a mathematical model which described the dynamical characteristic of autophagy induction controlled by mTORC1–AMPK-P–ULK1-P regulatory triangle with respect to various cellular stress (such as rapamycin or resveratrol treatment and starvation)^23^. In that model, according to the experimental data, double negative feedback loops are assumed between mTORC1–AMPK-P and mTORC1–ULK1-P. Besides, it has been already proved experimentally that active, phosphorylated AMPK is able to induce ULK1 via phosphorylation of its various Ser-residues (such as Ser-555, Ser-777)^25,38^, while ULK1-P kinase has a negative effect on AMPK activity^18,25^ generating an AMPK-P → ULK1-P ┤ AMPK-P negative feedback loop in the control network. Since the exact molecular mechanism of this network module is yet unknown, in our model a simple mathematical function was used to define the regulatory connection between AMPK-P and ULK1-P precisely. We claim that upon stress events (i.e. starvation or rapamycin treatment) these feedback loops of mTORC1–AMPK-P–ULK1-P regulatory triangle plays a crucial role in generating cellular autophagic response^23^.

In this study we reveal the dynamical characteristic of AMPK-P → ULK1-P ┤ AMPK-P negative feedback loop upon prolonged starvation or rapamycin treatment in human cell line by using both theoretical and molecular biological techniques. We suggest that mTORC1 down-regulation/AMPK up-regulation is repeated periodically in time resulting in an oscillatory characteristic for autophagy induction. We show that these features require a proper time delay in the AMPK-P → ULK1-P ┤ AMPK-P negative feedback loop. Therefore, we also predict various molecular mechanisms of this delay might to accomplish the precise oscillatory characteristic of the control network.

## Materials and methods

### Materials

Rapamycin (Sigma-Aldrich, R0395), DMEM—no glucose, no glutamine (Life Technologies, A14430-01), bafilomycin A1 (Sigma-Aldrich, M17931), were used for cellular treatments. All other chemicals were of reagent grade.

### Cell culture and maintenance

Human embryonic kidney (HEK293T, ATCC, CRL-3216) cell line was used as a model system. The cells were cultured in DMEM (Life Technologies, 41,965,039) media supplemented with 10% fetal bovine serum (Life Technologies, 10,500,064) and 1% antibiotics/antimycotics (Life Technologies, 15,240,062). The cell culture was maintained in a humidified incubator at 37 °C in 95% air and 5% CO_2_^39^.

### SDS-PAGE and Western blot analysis

Before treatments, the cells were synchronized by serum-free media. The treated cells were harvested and lysed with 20 mM Tris, 135 mM NaCl, 10% glycerol, 1% NP40, pH 6.8 for 30 min. Protein content of the cell lysates was measured with Pierce BCA Protein Assay (Thermo Scientific, 23,225)^39,40^. The samples were separated on 10% or 15% sodium dodecyl sulfate polyacrylamide gel and the SDS-PAGE was done by using Hoefer miniVE (Hoefer Inc.) Proteins were transferred to 0.45 µM PVDF membrane (Thermo Scientific, 88,518). The membranes were blocked with TBS Tween (0.1%), or 5% non-fat dry milk or 1% bovine serum albumin (Sigma-Aldrich, A9647)^39,40^. The membranes were incubated with the following antibodies: antiLC3B (SantaCruz, sc-271625), antip62 (Cell Signaling, 5114S), antiAMPK-P (Cell Signaling, 2531S), antiAMPK (Cell Signaling, 2603S), antip70S6K-P (Cell Signaling, 9234S), antip70S6K (SantaCruz, sc-8418), antiULK1-555-P (Cell Signaling, 5869S), antiULK1-777-P (Merck, ABC123), antiULK1 (Cell Signaling, 8054S) and antiGAPDH (Santa Cruz, 6C5), HRP conjugated secondary antibodies (Cell Signaling, 7074S, 7076S). The bands were visualized using a chemiluminescence detection kit (Thermo Fisher Scientific Inc., 32,106)^39,40^.

### Immunofluorescence

The cells were transferred to slides (CultureWell Chambered Coverglass for cell culture, Invitrogen, C37000). Cells were synchronized in serum free media and treated with rapamycin and bafilomycin A1. After that they were fixed in 4% paraformaldehyde for 15 min and washed with phosphate-buffered saline (PBS) three times. The permeabilizing of cells were performed with 0,25% Triton-X (in PBS) for 10 min and then cells were washed three times in PBS for 5 min. The cells were blocked and incubated with antip62 (Cell Signaling, 7695S) according to the antibody protocol. Cells were washed with PBS and incubated with Alexa Fluor 488 conjugated anti-rabbit (Cell Signaling, 4412S) for 1 h. After washing the nuclei were staining with DAPI (1:10,000) for 5 min and then the cells were washed again. The slides were mounted with FluorSave Reagent (Millipore, 345,789) and observed under a fluorescence microscope (Nikon Eclipse Ts2R)^23^. At each experimental step 4–6 different images were randomly photographed with similar settings. Total number of cells was counted by nuclei staining. The well demarcated, sharp p62 dots were counted in 80–100 cells using the program QuPath-0.2.1, which is freely available from https://github.com/qupath/qupath/releases/tag/v0.2.1.

### Mathematical modelling

The regulatory network was converted to a set of nonlinear ordinary differential equations (ODEs) and analysed using the techniques of dynamical system theory^41–43^. For details (i.e. for equations, codes and software) see Supplementary Information. Dynamical simulations were carried out using the program *XPPAUT,* which is freely available from https://www.math.pitt.edu/~bard/xpp/xpp.html^41,42^.

### Statistics

For densitometry analysis of Western blot data ImageJ software was used. The relative band densities were normalized to an appropriate total protein or GAPDH band used as reference protein (see Supplementary Information). For each of the experiments three independent measurements were carried out. Results are presented as mean values ± SD and were compared using ANOVA with Tukey’s multiple comparison post hoc test. Asterisks indicate statistically significant difference from the appropriate control: *p < 0.05; **p < 0.01^40^.

## Results

### Direct negative feedback loop between AMPK-P and ULK1-P results in homeostasis upon mTORC1 down-regulation

**Figure 1:**
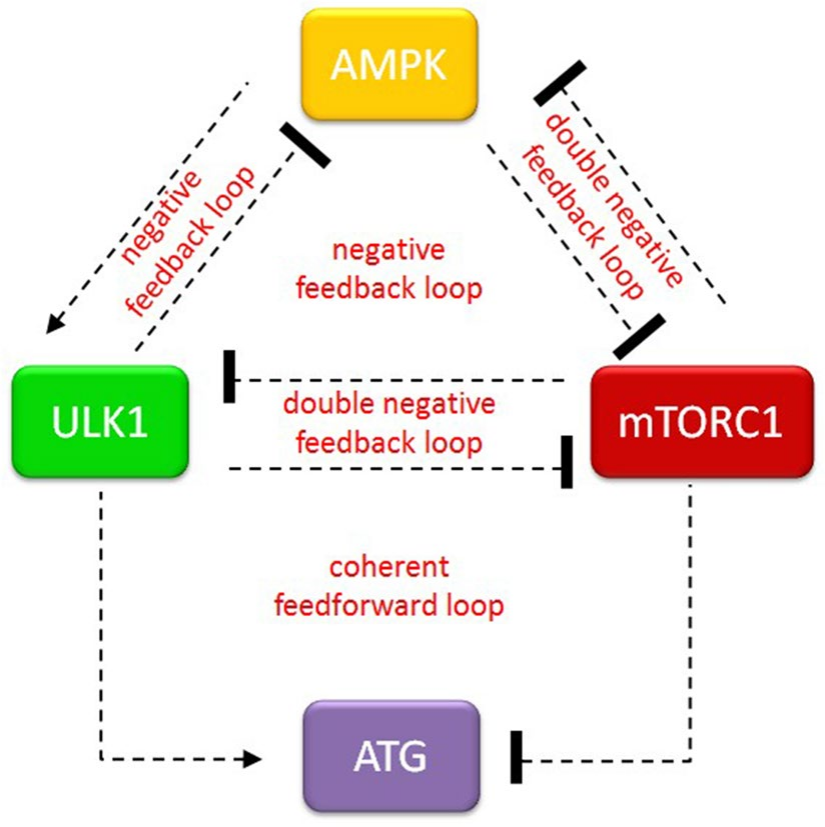
The wiring diagram of AMPK-P–mTORC1–ULK1-P regulatory triangle controlled stress response mechanism. The AMPK-P, mTORC1 and ULK1-P are denoted by isolated orange, red and green boxes, respectively. ATG (see purple box) refers to autophagy activator complex. Dashed line shows how the components can influence each other, while blocked end lines denote inhibition.

In this study we directly focus on AMPK-P → ULK1-P ┤ AMPK-P negative feedback loop to try to understand the dynamical features of this feedback loop in details by applying both molecular and theoretical biological techniques. Therefore, our previously published mathematical model was used for describing mTORC1–AMPK-P–ULK1-P controlled autophagy^23^, however first direct positive AMPK-P → ULK1-P and negative ULK1-P ┤ AMPK-P connections are assumed (see the wiring diagram on Fig. 1).

**Figure 2:**
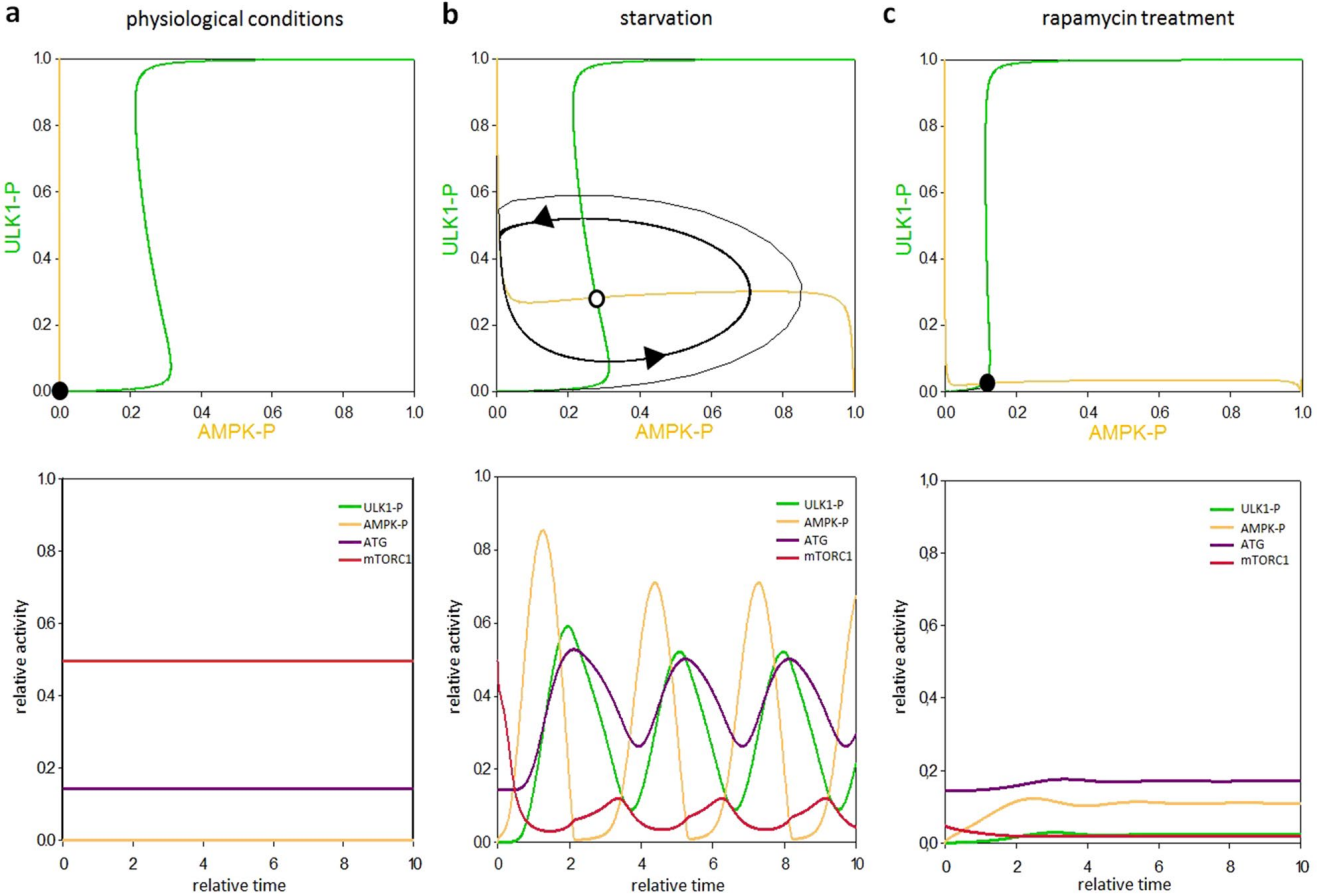
A simple negative feedback loop in the model results in a homeostatic behaviour during rapamycin treatment. The effect of direct AMPK-P → ULK1 ┤ AMPK-P negative feedback loop was systematically analysed upon various stress events. The balance curves of ULK1-P (green curve) and AMPK-P (orange curve) are plotted (upper panel). The phaseplanes are shown for (**a**) physiological conditions (**b**) starvation (STARV = 1.5) and (**c**) rapamycin treatment (mTORT = 0.1). Trajectories are depicted with black lines, while stable and unstable steady states are visualized with black and white dots, respectively. The computational simulations (lower panel) are determined upon (**a**) physiological conditions (**b**) starvation (STARV = 1.5) and (**c**) rapamycin treatment (mTORT = 0.1). The relative activity of mTORC1, AMPK-P, ULK1-P and autophagy activator complex (ATG) is shown.

To understand the consequence of this direct AMPK-P → ULK1-P ┤ AMPK-P feedback loop signal response curves were generated. In this case the dynamical characteristic of the non-linear differential equation system can be appropriately illustrated in a coordinated system spanned by AMPK-P and ULK1-P (Fig. 2, upper panel). If the other elements (i.e. mTORC1 and ATG, referring to autophagy activator complex) are in steady state, we can plot the balance curves for ULK1-P and AMPK-P (see the yellow and green curves on Fig. 2, upper panel). Along the balance curve, the rate of active, phosphorylated form is exactly balanced by its rate of inactive, de-phosphorylated form of the given component. The intersections between two balance curves are called equilibrium points: here the system has steady state solutions, representing the observable physiological states of the regulatory system. Under physiological conditions the balance curves intersect at close to zero, referring that both AMPK-P and ULK1-P are down-regulated (Fig. 2a, upper panel). Time course simulation shows that mTORC1 level is high, while autophagy has only a basal activity in this case (see relative activity of mTORC1 and ATG on Fig. 2a, lower panel). Nutrient deprivation is mimicked by increasing the activatory rate constant of AMPK-P. Depending on the parameter value this higher activity of AMPK-P generates a so called delayed negative feedback loop in AMPK-P–mTORC1–ULK1-P control network, which is able to accomplish a limit cycle oscillation (Fig. 2b). Namely, AMPK-P ┤ mTORC1 ┤ ULK1-P ┤AMPK-P feedback loop results in the periodic repeat of autophagy induction. Our computer simulation suggests that instead of stabilizing the self-cannibalism, the cell manages to generate a periodic repetition of this mechanism during starvation.

Rapamycin treatment was mimicked by suppressing the total level of mTORC1 (mTORT = 0.1), resulting in a diminish of AMPK-P ┤ mTORC1 ┤ ULK1-P ┤ AMPK feedback loop (Fig. 2c). In this case only AMPK-P → ULK1-P ┤ AMPK-P negative feedback loop remains in the control network. The direct connection between them results in a homeostatic response (Fig. 2c, upper panel). Namely, in the absence of mTORC1, AMPK-P quickly gets activated and turns on ULK1 by phosphorylation. ULK1-P blocks AMPK activation before it has no chance to induce autophagy, therefore no self-cannibalism is shown on the computer simulations (Fig. 2c, lower panel). However, this phenomenon is completely contradictory to previous experimental results, where rapamycin treatment induced an intense autophagy response^44^.

Our theoretical results suggest that a direct negative feedback loop between AMPK-P and ULK1-P cannot describe properly the stress response mechanism of the control network induced by well-known cellular stressors.

### Both starvation and rapamycin treatment generate a periodic repeat of autophagy

**Figure 3:**
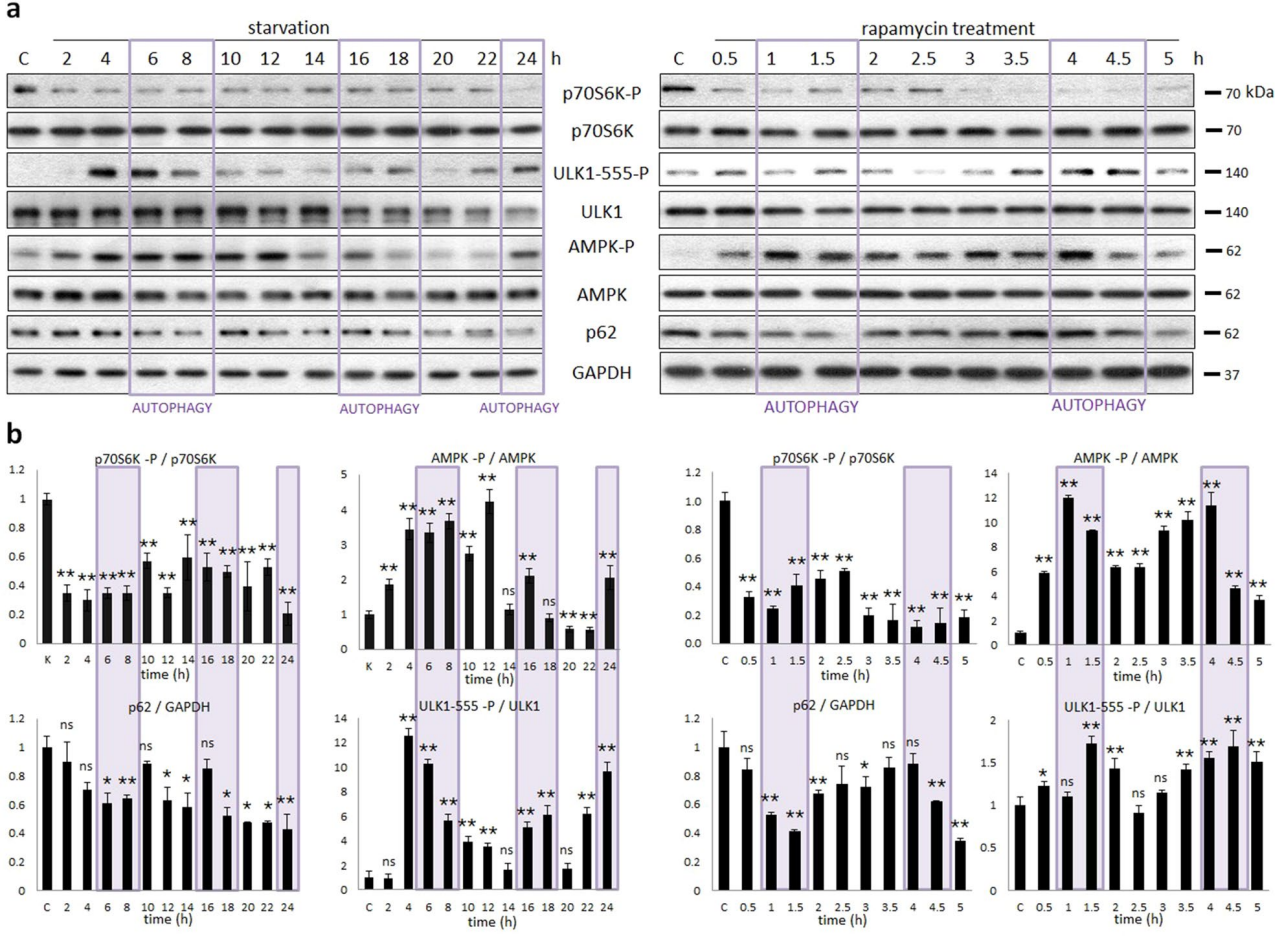
Prolonged starvation or rapamycin treatment results in oscillation of AMPK–mTORC1–ULK1 controlled autophagy. Starvation was induced in HEK293T cells by glucose depletion (**a**, panel left), while HEK293T cells were denoted in time after 100 nM rapamycin treatment. (**a**, panel right) The markers of autophagy (p62), AMPK-P, ULK1-P (ULK1-555-P) and mTORC1 (p70S6K-P) were followed by immunoblotting. GAPDH was used as loading control. (**b**) Densitometry data represent the intensity of p62 normalised for GAPDH, ULK1-555-P normalized for total level of ULK1, p70S6K-P normalized for total level of p70S6K and AMPK-P normalized for total level of AMPK. For each of the experiments, three independent measurements were carried out. Error bars represent standard deviation asterisks indicate statistically significant difference from the control: *p < 0.05; **p < 0.01.

Since our simplified model cannot explain the dynamical characteristic of rapamycin addition properly, we have to assume that a direct negative feedback connection between AMPK-P and ULK1-P is not correct to define autophagy induction. To build up a more precise mathematical model we re-investigate the time-dependency of prolonged nutrient depletion or rapamycin treatment experimentally (Fig. 3). HEK293T cells were synchronized, and then starvation (carbohydrate free medium, for 24 h) or rapamycin treatment (100 nM, for 5 h) was carried out. Samples were taken at specific time intervals (at every 2 h in case of starvation or every 30 min after rapamycin addition). The time-dependency of the key indicators of AMPK (phosphorylation status of AMPK), mTORC1 (phosphorylation of its target, p70S6K), ULK1 (phosphorylation status of Ser-555 residue of ULK1) and autophagy (p62) were detected by immunoblotting during treatments (Fig. 3).

mTORC1 quickly got down-regulated 30 min after rapamycin addition (see the dephosphorylation of p70S6K), while nutrient depletion also reduced mTORC1 activity significantly due to AMPK-P dependent inhibition (Fig. 3). Interestingly, upon starvation and rapamycin treatment the activation of most of the proteins has shown a rhythmic pattern. The activation profiles of both AMPK-P and ULK1-P change periodically generating a periodic activation of autophagy. Namely, the rhythmic pattern of p62 level precisely suggests that autophagic response is also oscillating upon permanent starvation or rapamycin-dependent mTORC1 inhibition. These proteins seem to have a period of approximately 8 h during starvation and 2 h upon rapamycin treatment. Since the periodic change of autophagy could be due to cell cycle or circadian rhythm, therefore rapamycin treatment was repeated in non-synchronized cell population (see Supplementary Fig. 1). The periodic activation of autophagy was clearly observed suggesting that the oscillatory pattern of autophagy is due to the self-cannibalism itself.

**Figure 4:**
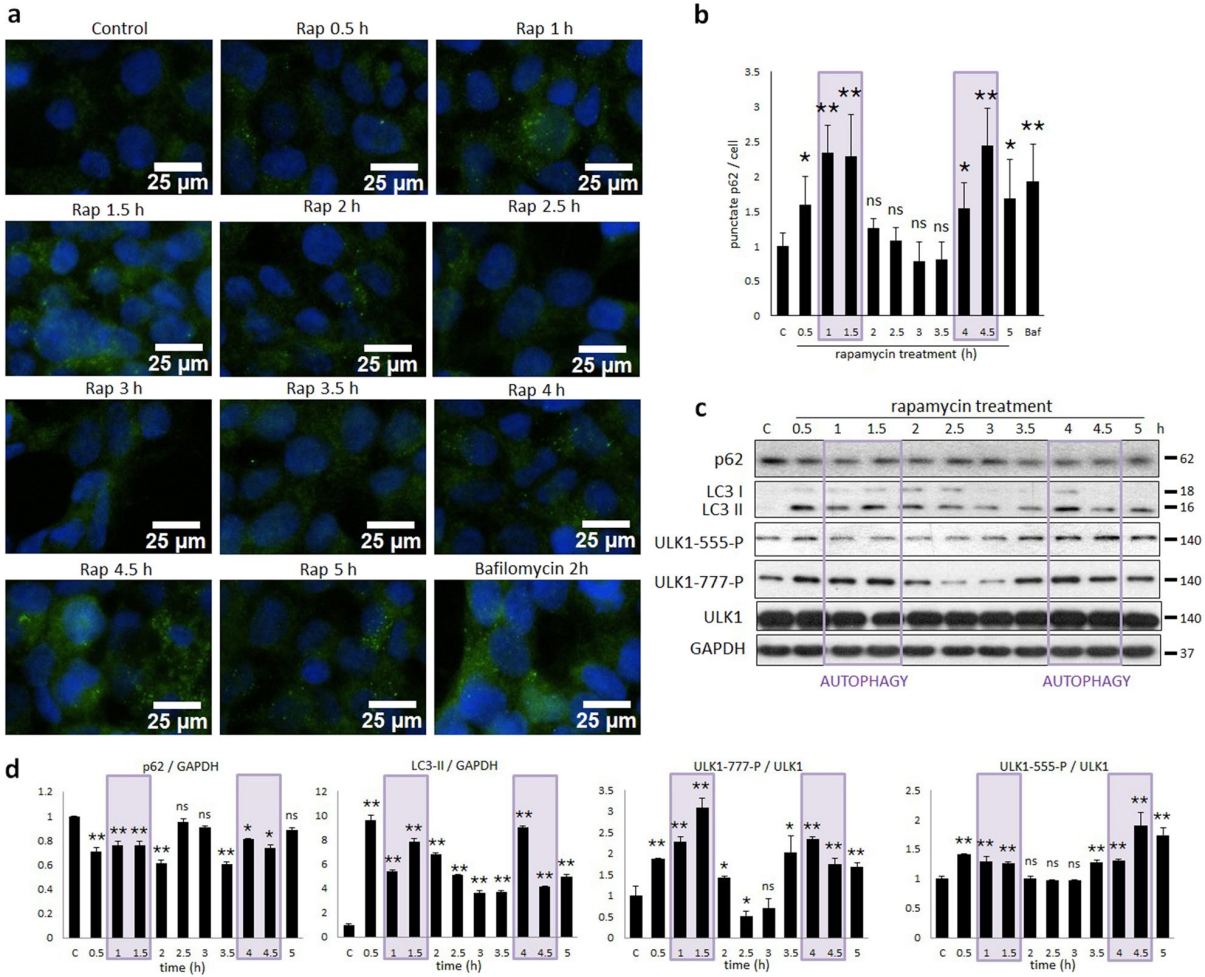
Prolonged rapamycin treatment results in oscillation of AMPK–mTORC1–ULK1 controlled autophagy. HEK293T cells were denoted in time after 100 nM rapamycin treatment. (**a**) Autophagy activation was checked by immunofluorescence microscopy. p62 was stained by green fluorescence dye. Bafilomycin A1 (100 nM, 2 h) was used as positive control. (**b**) Quantification and statistical analysis of immunofluorescence microscopy data. Error bars represent standard deviation, asterisks indicate statistically significant difference from the control: *p < 0.05; **p < 0.01. (**c**) The markers of autophagy (LC3, p62) and ULK1-P (ULK1-555-P, ULK1-777-P) were followed by immunoblotting. GAPDH was used as loading control. (**d**) Densitometry data represent the intensity of p62 and LC3II normalised for GAPDH, ULK1-555-P and ULK1-777-P normalized for total level of ULK1. For each of the experiments, three independent measurements were carried out. Error bars represent standard deviation, asterisks indicate statistically significant difference from the control: *p < 0.05; **p < 0.01.

To further confirm the periodic activation of autophagy upon the above mentioned prolonged rapamycin treatment autophagy was also detected by using immunofluorescence microscopy (Fig. 4a,b). In these experiments p62 was stained by green fluorescence dye. As positive controls, bafilomycin A1 (100 nM, 2 h) was used. The increase of relative amount of autophagosomes has shown a periodic pattern when mTOR was inhibited (see the sharp green dots on Fig. 4a). Namely, more than a two-fold increase in levels was observed afterboth one and four hours of rapamycin treatment (Fig. 4b). In both cases, the amount of autophagosomes remained large for approximately 30 min long. Then its level always dropped significantly and became similar to its basal level (Fig. 4a,b). Corresponding to the periodic repeat of the relative amount of autophagosomes the densitometry data of autophagy markers (p62, LC3II) changed similarly (Fig. 4c,d). Namely, both the decrease of p62 level and increase of LC3II/GAPDH ratio were observed in every one and a half hour further suggesting that autophagy worked properly.

With our data we first demonstrate that sustained mTORC1 down-regulation via starvation or rapamycin addition results in a periodic repeat of autophagy induction.

### The possible regulatory ways to manage a delayed negative feedback loop between AMPK-P and ULK1-P

Kinetic models have already proved that negative feedback loop can generate a sustained oscillation in molecular systems if (1) negative feedback is present, (2) the negative feedback is sufficiently delayed, (3) the kinetic rate laws are sufficiently “nonlinear” and (4) the reactions occur in appropriate time scales^45^. Therefore, a certain delay mechanism has to be built in the AMPK-P → ULK1-P positive arm or the ULK1-P ┤ AMPK-P negative arm by containing some delaying effect compared to the other one.

Since our experimental data suggest that AMPK phosphorylation always precedes ULK1-P (Fig. 3) we suppose that the “delaying effect” is somehow built in the AMPK-P → ULK1-P positive arm of AMPK-P–ULK1-P negative feedback loop. First we tried to explore the possible delaying effects on AMPK-P → ULK1-P positive arm theoretically.

**Figure 5:**
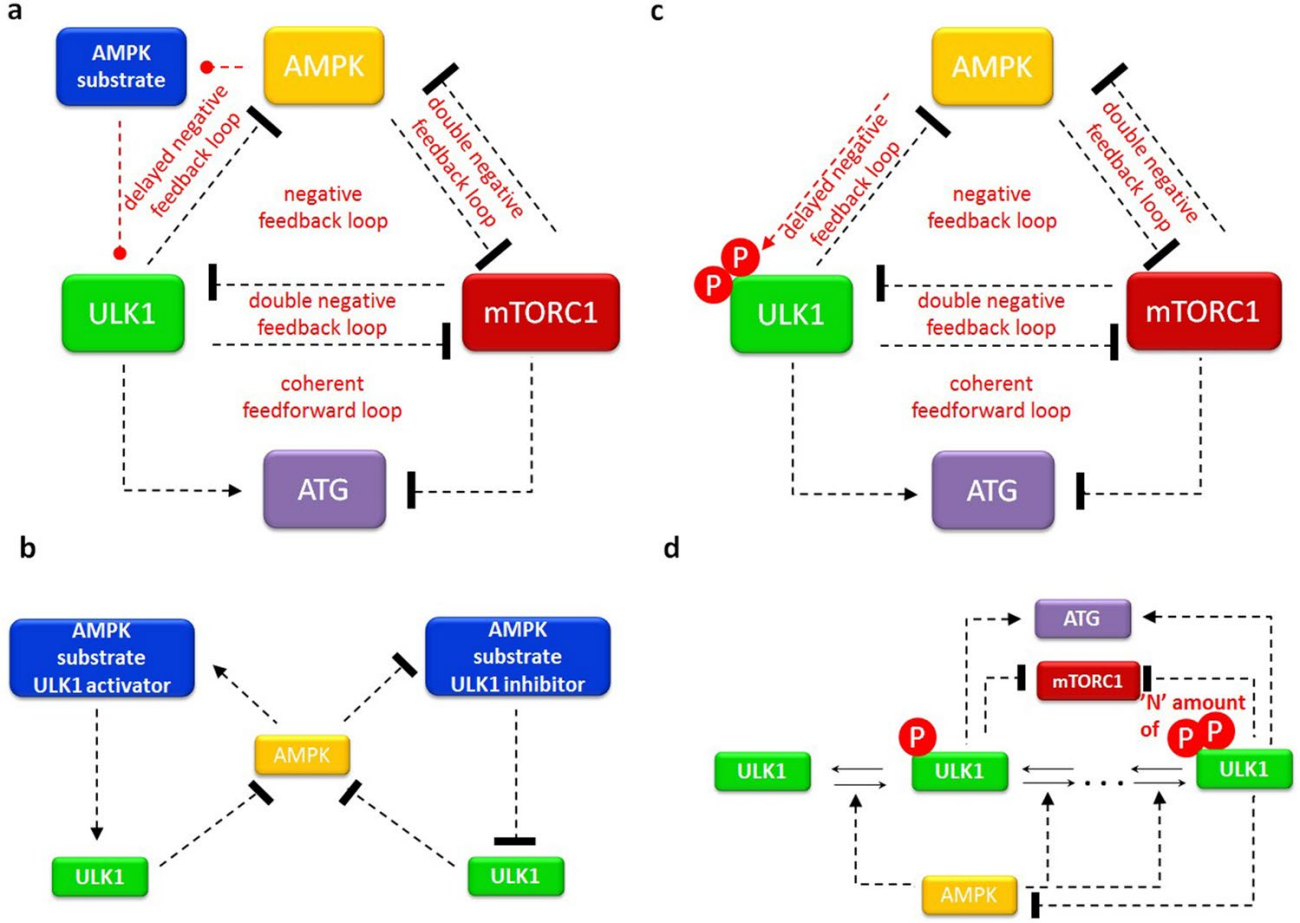
The possible mechanism of generating delayed AMPK-P–ULK1-P negative feedback loop. The wiring diagram of AMPK-P–mTORC1–ULK1-P regulatory triangle (**a**,**c**) and the detailed mechanism or delayed negative feedback loop between AMPK-P and ULK1-P (**b**,**d**) are plotted when the delay on ULK1-P activation is achieved by (**a**,**b**) an extra regulatory element or (**c**,**d**) multi-phosphorylation of ULK1. The AMPK-P, mTORC1 and ULK1-P are denoted by isolated orange, red and green boxes, respectively. ATG (see purple box) refers to autophagy activator complex. Dashed line shows how the components can influence each other, while blocked end lines denote inhibition.

In biological protein–protein networks this “delaying effect” usually is performed by inserting an extra molecular element in the control system (Fig. 5a,b). In this case, ULK1 is not directly phosphorylated by AMPK-P, rather through an AMPK substrate, while this substrate can change the activity of ULK1. Theoretically both AMPK-P ┤ AMPK substrate ┤ ULK1-P and AMPK-P → AMPK substrate → ULK1-P connections are possible. The only criterion is that the signs have to be the same through the regulatory arm to guarantee the negative feedback loop between ULK1-P and AMPK-P in the control network.

We can also assume that the regulation is somehow a multistep process generating intermediate components (Fig. 5c,d). Most of the proteins have more than one residues which can be phosphorylated by another molecule (i.e. ULK1 has more than one AMPK-dependent phosphorylation site). Therefore, besides Ser-555 on ULK1 we checked whether AMPK-dependent Ser-777 phosphorylation has any role during rapamycin treatment (Fig. 4c,d). Since Ser-777 phosphorylation comes earlier than Ser-555 phosphorylation, we can assume that AMPK-P can phosphorylate ULK1 sequentially upon mTORC1 inhibition. These phosphorylation steps form variously phosphorylated ULK1 molecules with various activities (Fig. 5c,d), therefore this multiphosphorylation might generate a proper “delay” in the negative feedback loop essential for sustained oscillation.

We cannot rule out that there is a delay on ULK1-P ┤ AMPK-P, as well, although AMPK-P de-phosphorylation quickly follows ULK1 activation. For simplicity we assume there only one arm is delayed and this is AMPK-P → ULK1-P.

### Delayed negative feedback between AMPK-P and ULK1-P results in limit cycle oscillation with respect to mTORC1 down-regulation

**Figure 6:**
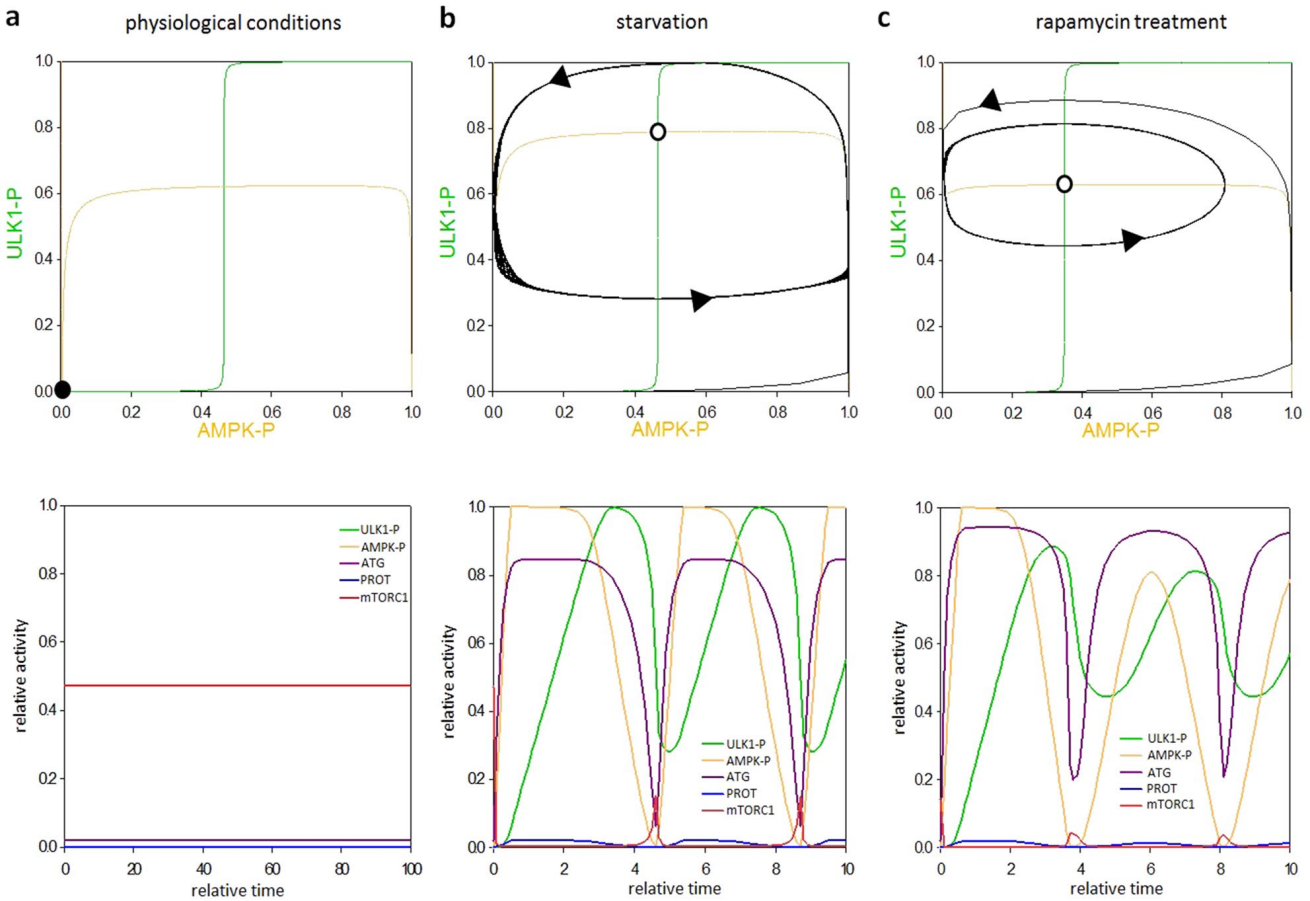
An extra element in the AMPK-P–ULK1-P negative feedback loop results in an oscillatory characteristic both in starvation and rapamycin treatment. The effect of delayed AMPK-P → AMPK substrate → ULK1-P ┤ AMPK-P negative feedback loop was systematically analysed upon various stress events. The balance curves of ULK1-P (green curve) and AMPK-P (orange curve) are plotted (upper panel). The phaseplanes are shown for (**a**) physiological conditions (**b**) starvation (STARV = 0.5) and (**c**) rapamycin treatment (mTORT = 0.3). Trajectories are depicted with black lines, while stable and unstable steady states are visualized with black and white dots, respectively. The computational simulations (lower panel) are determined upon (**a**) physiological conditions (**b**) starvation (STARV = 0.5) and (**c**) rapamycin treatment (mTORT = 0.3). The relative activity of mTORC1, AMPK-P, ULK1-P, PROT (AMPK substrate) and autophagy activator complex (ATG) is shown.

**Figure 7:**
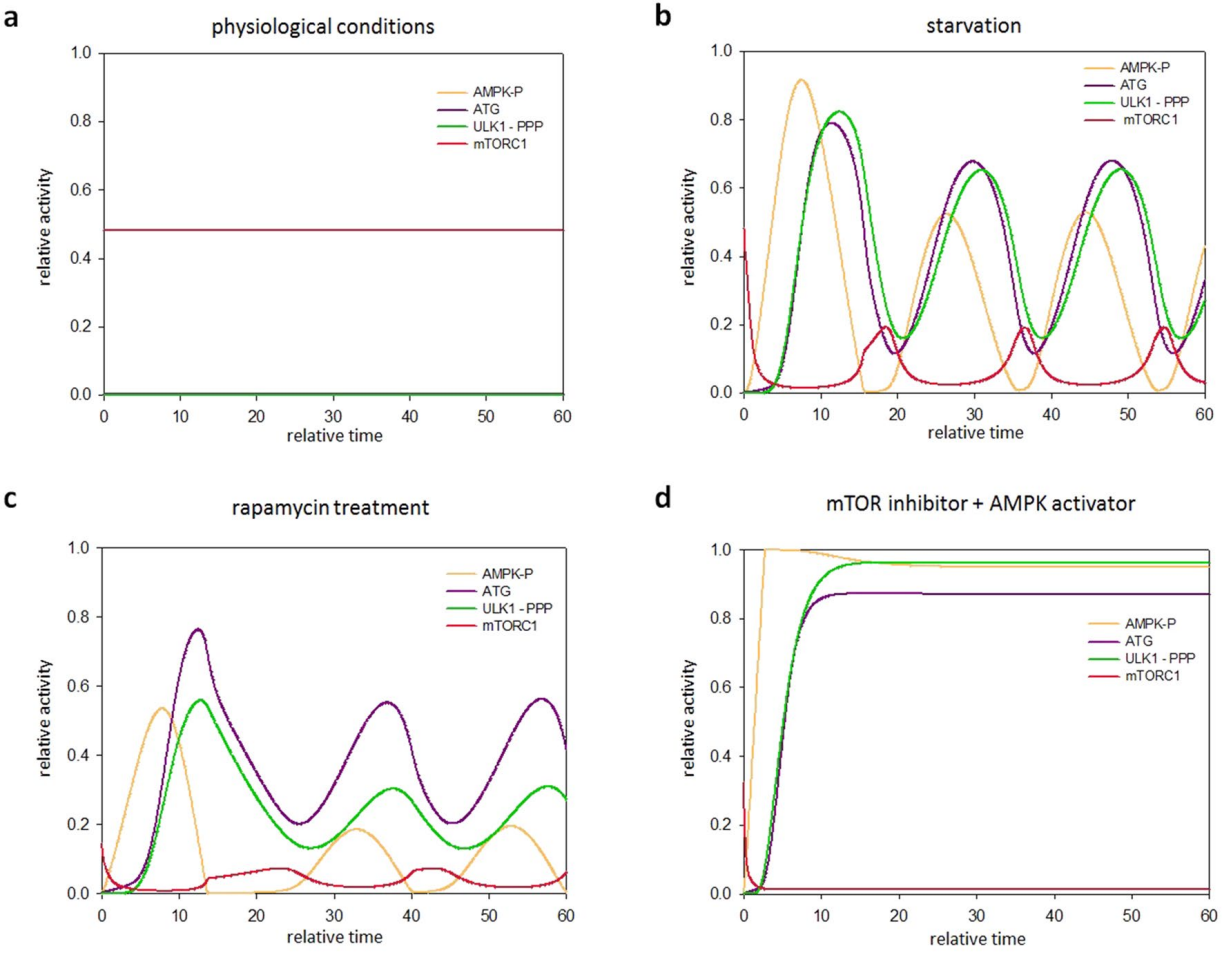
The multi-phoshporylation of ULK1 results in an oscillatory characteristic both in starvation and rapamycin treatment. The effect of delayed AMPK-P → ULK1-P → ULK1-PP → ULK1-PPP ┤ AMPK-P negative feedback loop was systematically analysed upon various stress events. The computational simulations are determined upon (**a**) physiological conditions, (**b**) starvation (STARV = 0.1), (**c**) rapamycin treatment (mTORT = 0.3) and (**d**) when mTORC1 inhibition is combined with AMPK-P activation (mTORT = 0.3, kaak = 0.4). The relative activity of mTORC1, AMPK-P, ULK1-PPP (the active form of ULK1) and autophagy activator complex (ATG) is shown.

To further test the oscillatory characteristic of the above mentioned delayed negative feedback loop computer simulations were carried out. Our simple model of ULK1-P-AMPK-P negative feedback exhibits limit cycle oscillation if there is a sufficiently long time delay in the regulatory loop managed by series of intermediates via an extra AMPK substrate (Fig. 6) or multiphosphorylation of ULK1 (Fig. 7).

In Fig. 6 upper panel we plot the balance curve of nonlinear differential equations of ULK1-P and AMPK-P. At physiological conditions there is one steady state in the system, with low level of both ULK1-P and AMPK-P suggesting that autophagy is not active (Fig. 6a, upper panel). In case of starvation the kink in the nullclines forces the dynamical system to overshoot and undershoot the steady state repeatedly generating a sustained oscillation (Fig. 6b, upper panel). In addition, when mTORC1 is fully down-regulated by rapamycin treatment the control network executes a limit cycle oscillation due to the delayed negative feedback loop between ULK1-P and AMPK-P (Fig. 6c, upper panel). Time series data further confirmed that sustained oscillation is nicely achieved by building in an intermediate element in the negative feedback loop of ULK1-P and AMPK-P (Fig. 6b,c, lower panel).

Similar results were detected when time delay was achieved via multiphosphorylation of ULK1 (Fig. 7b,c). Recently we have also showed that various mTORC1 inhibitors and/or AMPK activators (i.e. resveratrol or EGCG) induced stable autophagy and no oscillation was observed^46,47^. Our computation simulation further confirms that the control network turns on autophagy when mTORC1 down-regulated and AMPK hyper-activated at the same time (Fig. 7d). In this case the ULK1-P-AMPK-P negative feedback loop is not properly balanced anymore. Namely, ULK1-P is not able to win against the high level of AMPK-P. Although ULK1-P level gets high, AMPK-P also remains active, resulting in a permanent autophagy.

Our model suggests that limit cycle oscillation of autophagic response in case of nutrient depletion or starvation requires a delayed negative feedback loop between ULK1-P and AMPK-P, but it also assumes appropriate AMPK-P and mTORC1 levels in the cell.

## Discussion

Cellular homeostasis is crucial for the dynamic changes required for the cell to respond various stimuli (such as alteration of nutrient availability). The evolutionary conserved self-cannibalism, called autophagy, has an essential role in digesting damaged or unnecessary components of the cell. Applying both experimental and theoretical methods recently we have analysed the dynamical features of stress response mechanism inducing autophagy by re-wiring a control network built by the two sensors of nutrient conditions (i.e. AMPK and mTORC1) and the key component of autophagy inducer, called ULK1. We claimed that AMPK-P–mTORC1 and mTORC1–ULK1-P double negative feedback loops are required for the switch-like characteristic of the system^23^. However, we have not analysed the importance of the negative feedback loops of mTORC1–AMPK-P–ULK1-P controlled network yet (Fig. 1). Two negative feedback loops can be found in the regulatory system, namely AMPK-P → ULK1-P ┤AMPK-P and AMPK-P ┤ mTORC1 ┤ ULK1-P ┤ AMPK-P, respectively (Fig. 1).

Assuming simple direct regulatory connections between the elements our theoretical analysis suggested that due to the nutrient deprivation increased AMPK-P level the AMPK-P ┤ mTORC1 ┤ ULK1-P ┤ AMPK-P negative feedback loop can generate a sustained oscillation of autophagy induction (Fig. 2b). Our experimental data also confirmed that starvation resulted in periodic repeat of a physiological state (with high level of mTORC1, low levels of AMPK-P and ULK1-P) and an autophagic state (with low level of mTORC1, high levels of AMPK-P and ULK1-P) with around eight-hour periods upon twenty-four hours of treatment (Fig. 3). Although the periodic repeat of ULK-1 level has been already described by Nazio et al.^36,48^, this is the first time when sustained oscillation of autophagy was detected during glucose starvation.

Interestingly, in some studies AMPK phosphorylation was detected upon rapamycin-dependent down-regulation of mTORC1^23,31^, while other scientists were not able to detect AMPK activation during rapamycin treatment^32,33^. However, in these cases samples were taken only at the end of treatment. Therefore, to further explore rapamycin addition, a five hours long treatment was carried out and samples were taken in every 30 min. We observed the oscillation of autophagy process together with AMPK phosphorylation after addition of rapamycin, and the period time was two and a half hours long (Figs. 3, 4). Our results might give a good explanation for the contradictory results in the literature.

Since mTORC1 activity got significantly decreased during prolonged rapamycin treatment and starvation, the AMPK-P ┤ mTORC1 ┤ ULK1-P ┤ AMPK-P negative feedback loop diminished from the control network suggesting that the oscillatory characteristic required another negative feedback loop upon down-regulation of mTORC1. Therefore, we supposed that the periodic repeat of a physiological state (with low levels of AMPK-P and ULK1-P) and an autophagic state (with high levels of AMPK-P and ULK1-P) is achieved via AMPK-P → ULK1-P ┤AMPK-P. However, the regulatory system generates a homeostatic response and no proper autophagy induction is detected simulating rapamycin treatment when direct connections were assumed in AMPK-P–ULK1-P negative feedback loop (Fig. 2c). Kinetic analysis has been already suggested, that a so called time-delay is essential for a proper oscillation. Since induction of AMPK-P clearly preceded ULK1 phosphorylation (Fig. 3) we assumed that this time-delay was built in AMPK-P → ULK1-P arm of the negative feedback loop.

By using theoretical analysis, we investigated various biological relevant options to generate a proper time delay in the control network. The two most possible scenario were the incorporation of an extra regulatory element on AMPK-P → ULK1-P arm or the AMPK-P-dependent multiphoshorylation of ULK1 (Fig. 5).

To identify an extra regulatory element between AMPK-P and ULK1-P, calling AMPK substrate, the BioGrid, the DIP, the MINT, the InnateDB and the IntAct online freely available databases were used. Using these databases all the possible ULK1 interactors were collected (for details see the Supplementary Information). Since AMPK-P, as a Ser/Thr protein kinase, controlling its targets via phosphorylation, we supposed that the active AMPK-P regulated the ULK1 interactors via phosphorylation. Therefore we identified potential Ser and Thr phosphorylation sites on ULK1 interactors with Group-based Prediction System 5.0^49^ and we verified the results by NetPhos 3.1, a freely available software^50^. We found that many of the ULK1 interactors (such as PP1CA, PP2A, NEDD4L) contain one or more consensus phosphorylation motifs of AMPK-P (for details see the Supplementary Information, Supplementary Table S1). This analysis suggested that AMPK-P might be able to promote ULK1 activation indirectly throughout its interactors, however, these connections later must be proven experimentally. Since AMPK-P has to have a positive effect on ULK1 activity, for the proper operation of the regulatory arm only AMPK-P → AMPK substrate → ULK1-P and AMPK-P ┤ AMPK substrate ┤ ULK1-P connections are possible, however the exact sign of the relationships needs to be verified later. Note here, that we cannot rule out the importance of the direct AMPK-P → ULK1-P connection via AMPK-P-dependent ULK1 phosphorylation (i.e. ULK1-555 and ULK1-777 phosphorylation by AMPK-P), but we claim that this indirect connection might be essential to explain the oscillatory characteristic of stress response mechanism upon rapamycin treatment.

According to our molecular biological knowledge, introducing time delay on the AMPK-P–ULK1-P negative feedback loop, a theory where AMPK-P is able to multiphosphorylate ULK1 is also likely. It has already shown that AMPK-P was able to phosphorylate ULK1 on its following residues: Ser-317, Ser-467, Ser-555, Thr-574 and Ser-777^25,38^. Besides, using the AMPK-P consensus phosphorylation motif another Ser and Thr phosphorylation sites on ULK1 were detected by using Group-based Prediction System 5.0 and checked with NetPhos 3.1 (for details see the Supplementary Information, Supplementary Table S2). Both the AMPK-P-dependency of these ULK1 phosphorylation sites and the sign (whether its positive or negative) of these phosphorylations have to be clarified later experimentally. We have already suggested that multiphosphorylation is able to introduce an appropriate time delay in a regulatory network^51^. Here we confirm that a proper oscillation of autophagic response is clearly manageable by assuming AMPK-P-dependent multiphosphorylation of ULK1 (Fig. 7).

Question immediately arises, namely what is the biological importance of periodic autophagy induction upon intermediate level of cellular stress. We assume that with this periodic change of autophagy during starvation or rapamycin treatment the system might have an opportunity to utilize the building blocks produced from more complex biological elements via autophagy.

Our theoretical analysis has revealed that stress level has to reach an intermediate level for oscillation of autophagy (data not shown). While low level of stress did not increase significantly the basal level of autophagic flux, excessive level of cellular stress induced cell death later. We claim that the oscillation of autophagy at intermediate level of cellular stress (such as prolonged rapamycin treatment or starvation) is essential to remove the damaged elements and utilize the unnecessary components, otherwise the cell has to commit early cell death.

To explore how the regulation of the cellular survival processes are achieved with precise molecular balance of mTORC1-AMPK upon autophagy, has a great importance in several cellular stress related diseases such as neurodegenerative diseases (e.g. Parkinson’s disease, Alzheimer’s disease), metabolic diseases, inflammation and carcinogenesis^52^. Our systems biological approach improves the understanding of the molecular basis of these complex syndromes and might help to promote future therapeutic technics against these diseases, too.

## Supplementary information

Supplementary Information.

Supplementary Table 1.

Supplementary Table 2.

## Abbreviations

mTOR: Mammalian target of rapamycin
AMPK: 5′ AMP-activated protein kinase
Rap: Rapamycin
