# Supplementary material for: Fine-tuning of AMPK–ULK1–mTORC1 regulatory triangle is crucial for autophagy oscillation
Source: Sci Rep. 2020 Oct 20;10:17803. doi: 10.1038/s41598-020-75030-8 (PMC7576158; doi:10.1038/s41598-020-75030-8)
Supplement: Supplementary file 1 — Supplementary Information. [file 41598_2020_75030_MOESM1_ESM.pdf]

Supplementary Material

"Fine-tuning of AMPK-ULK1-mTORC1 regulatory triangle is crucial for autophagy oscillation"

Marianna Holczer[1], Bence Hajdú[1], Tamás Lőrincz [2], András Szarka [2], Gábor Bánhegyi[1,3], Orsolya Kapuy[1,*]

[1] Semmelweis University, Department of Medical Chemistry, Molecular Biology and Pathobiochemistry, Budapest, Hungary

[2] Budapest University of Technology and Economics, Laboratory of Biochemistry and Molecular Biology, Department of Applied Biotechnology and Food Science, Budapest, Hungary

[3] Pathobiochemistry Research Group of the Hungarian Academy of Sciences and Semmelweis University, Budapest, Hungary

I. Describing the theoretical analysis

In this section, we briefly describe the mathematical approach used to study the autophagy induction. A system level view can be developed by bringing together the components and interactions reported in the literature. Such a network can be translated into a set of mathematical equations that describe how each component concentration/activity in the network changes with the time. The rate of change of a component is described by ordinary differential equation (ODE) based on biochemical reaction kinetics (see equation below). Each biochemical reaction is represented as a term on the right hand side of the ODE for a component participating in the reaction [1,2]. Each reaction in the network can be described either by using law of mass action or Michaelis-Menten kinetics [3-5].

A generic differential equation describing the temporal changes of protein X_a is composed of two parts: production and consumption terms.

$$dX_a/dt = k_s + k_{act}*(X_T-X_a) - (k_d + k_{in})*X_a$$

Where:

X_a – concentration of active X	k_{act} – activation rate constant of X_a
X_T – total concentration of X	k_d – degradation rate constant of X
k_s – synthesis rate constant of X	k_{in} – inactivation rate constant of X_a

The production can be given by protein synthesis and/or an activation term, while the consumption can be given by protein degradation and/or inactivation term. Usually synthesis, degradation, binding and dissociation reactions are described by mass action kinetics, whereas protein activity can be described either by mass action or Michaelis-Menten kinetics [3, 6]. For example, if the activity of protein is controlled by covalent modification involving multi-site phosphorylations, Michaelis-Menten kinetics provides a good approximation for the process [7, 8]. The value of parameters (rate constants, Michaelis constants) and initial conditions have to be specified in order to solve ODEs. The non-linear nature of biological processes makes it difficult to find the solution of ODEs analytically hence the equations has to be solved numerically. The equations can be solved using different numerical integration methods that are implemented as solvers in many of the freely available computer software.

Solving a set of non-linear ODEs gives the time evolution of the protein concentration/activity called **time courses**. Further, ODEs can be solved to obtain the input-output relationship called as **signal response curves** or as **one parameter bifurcation diagram** [2, 3, 9]. An input is the signal strength that is varied to obtain the steady state behaviour of the control system. This helps to capture the qualitative changes in the behaviour of the system. For example, the system behaviour can become abrupt and discontinuous when signal strength is increased

from a low value to high value. A point at which such a qualitative change in the system occurs is defined as bifurcation point [2].

In this work, the temporal profiles and signal response curves were computed numerical using *XPP-AUT*. All the simulations presented in the text are based on the following XPP codes. The rate constants (k) have the dimension of min^{-1} and Michaelis constants (*J*) are dimensionless. The proteins levels/activities are given in arbitrary units (a.u). The starting parameter set was able to refer to physiological conditions. The parameters values were perturbed to capture all the possible qualitative behaviours that the given network can exhibit.

Simulating AMPK-ULK1-mTORC1 regulatory triangle when a direct negative feedback loop is present between AMPK and ULK1

The detailed description of the elements of the theoretical models

	description
ULK1	the active form of ULK1
ULK1T	total level of ULK1
AMPK	the active form of AMPK
AMPKT	total level of AMPK
mTOR	the active form of mTORC1
mTORT	total level of mTORC1
ATG	the active autophagy activator complex
STARV	level of starvation

The detailed description of the constants of the theoretical models

	description
kaulk	background activation of ULK1
kaulk'	AMPK-dependent activation of ULK1
kiulk	background inactivation of ULK1
kiulk'	mTORC1-dependent inactivation of ULK1
Julk	Michaelis-constant of ULK1
kaak	background activation of AMPK
kiak	background inactivation of AMPK
kiak'	ULK1-dependent inactivation of AMPK
kiak"	mTORC1-dependent inactivation of AMPK
Jampk	Michaelis-constant of AMPK
kamtor	background activation of mTORC1
kimtor	background inactivation of mTORC1
kimtor'	AMPK-dependent inactivation of mTORC1
kimtor"	ULK1-dependent inactivation of mTORC1
Jmtor	Michaelis-constant of mTORC1
kaau	background activation of autophagy
kaau'	ULK1-dependent activation of autophagy
kiau	background inactivation of autophagy
kiau'	mTORC1-dependent inactivation of autophagy

The code for simulating signal response curves

```
# a model to generate signal response curves when a direct negative
feedback loop is present between AMPK and ULK1

# initial conditions
init AMPK=0, ULK1=0

# differential equtaions
# ULK1 represents the active form of ULK1
ULK1' = (kaulk  + kaulk'*AMPK)*(ULK1T-ULK1)/(Julk + ULK1T-ULK1) - (kiulk +
kiulk'*mTOR)*ULK1/(Julk + ULK1)

# AMPK represents the active form of AMPK
AMPK' = (kaak + STARV)*(AMPKT-AMPK)/(Jampk + AMPKT-AMPK) - (kiak +
kiak'*ULK1 + kiak"*mTOR)*AMPK/(Jampk + AMPK)

# steady state function
# mTOR represents the active form of mTORC1
mTOR = kamtor*mTORT/(kamtor + kimtor + kimtor'*AMPK + kimtor"*ULK1)

# parameters
# to simulate rapamycin treatment: mTORT = 0.1
# to simulate starvation: STARV = 1.5
p STARV=0
p kaulk=0.01, kaulk'=1, kiulk=0.1, kiulk'=3, Julk=0.01, ULK1T=1
p kaak=0.3, kiak=0.1, kiak'=5, kiak"=5, AMPKT=1, Jampk=0.005
p kamtor=0.01, kimtor=0.01, kimtor'=0.25, kimtor"=0.25, Jmtor=0.01, mTORT=1

done
```

The code for time course simulations

```
# a model to simulate time courses when a direct negative feedback loop is
present between AMPK and ULK1

# initial conditions
init ULK1=0.00001, AMPK=0.00065, ATG=0.14287

# differential equations
# ULK1 represents the active form of ULK1
ULK1' = (kaulk  + kaulk'*AMPK)*(ULK1T-ULK1)/(Julk + ULK1T-ULK1) - (kiulk +
kiulk'*mTOR)*ULK1/(Julk + ULK1)

# AMPK represents the active form of AMPK
AMPK'  =  (kaak  +  STARV)*(AMPKT-AMPK)/(Jampk  +  AMPKT-AMPK)  -  (kiak  +
kiak'*ULK1 + kiak"*mTOR)*AMPK/(Jampk + AMPK)

# ATG represents the active form of autophagy activator complex
ATG' = (kaau + kaau'*ULK1)*(1-ATG) - (kiau + kiau'*mTOR)*ATG

# steady state function
# mTOR represents the active form of mTORC1
mTOR = kamtor*mTORT/(kamtor + kimtor + kimtor'*AMPK + kimtor"*ULK1)

# parameters
# to simulate rapamycin treatment: mTORT = 0.1
# to simulate starvation: STARV = 1.5
p STARV=0
p kaulk=0.01, kaulk'=1, kiulk=0.1, kiulk'=3, Julk=0.01, ULK1T=1
p kaak=0.3, kiak=0.1, kiak'=5, kiak"=5, AMPKT=1, Jampk=0.005
p kamtor=0.01, kimtor=0.01, kimtor'=0.25, kimtor"=0.25, Jmtor=0.01, mTORT=1
p kaau=0.01, kaau'=0.1, kiau=0.01, kiau'=0.1

done
```

Simulating AMPK-ULK1-mTORC1 regulatory triangle when AMPK can induce ULK1 throughout an extra protein

The detailed description of the elements of the theoretical models

	description
ULK1	the active form of ULK1
ULK1T	total level of ULK1
AMPK	the active form of AMPK
AMPKT	total level of AMPK
mTOR	the active form of mTORC1
mTORT	total level of mTORC1
Prot	the active form of AMPK-activated protein
ATG	the active autophagy activator complex
STARV	level of starvation

The detailed description of the constants of the theoretical models

	description
kaulk	background activation of ULK1
kaulk'	Prot-dependent activation of ULK1
kiulk	background inactivation of ULK1
kiulk'	mTORC1-dependent inactivation of ULK1
Julk	Michaelis-constant of ULK1
kaak	background activation of AMPK
kiak	background inactivation of AMPK
kiak'	ULK1-dependent inactivation of AMPK
kiak"	mTORC1-dependent inactivation of AMPK
Jampk	Michaelis-constant of AMPK
kamtor	background activation of mTORC1
kimtor	background inactivation of mTORC1
kimtor'	AMPK-dependent inactivation of mTORC1
kimtor"	ULK1-dependent inactivation of mTORC1
Jmtor	Michaelis-constant of mTORC1
kapr	background activation of Prot
kapr'	AMPK-dependent activation of Prot
kipr	background inactivation of Prot
Jpr	Michaelis-constant of Prot
kaau	background activation of autophagy
kaau'	ULK1-dependent activation of autophagy
kiau	background inactivation of autophagy
kiau'	mTORC1-dependent inactivation of autophagy

The code for simulating signal response curves

```
# a model to simulate signal response curve when AMPK induces ULK1
throughout an extra protein

# initial conditions
init ULK1=0, AMPK=0

# differential equtaions
# ULK1 represents the active form of ULK1
ULK1' = (kaulk + kaulk'*Prot)*(ULK1T-ULK1)/(Julk + ULK1T-ULK1) - (kiulk +
kiulk'*mTOR)*ULK1/(Julk + ULK1)

# AMPK represents the active form of AMPK
AMPK' = (kaak + STARV)*(AMPKT-AMPK)/(Jampk + AMPKT-AMPK) - (kiak +
kiak'*ULK1 + kiak"*mTOR)*AMPK/(Jampk + AMPK)

# steady state function
# mTOR represents the active form of mTORC1
mTOR = kamtor*mTORT/(kamtor + kimtor + kimtor'*AMPK + kimtor"*ULK1)

# Prot represents the active form of extra protein
Prot = GK(kapr + kapr'*AMPK,kipr,Jpr,Jpr)
```

```
# 'Goldbeter-Koshand' function (GK)
GB(arg1,arg2,arg3,arg4) = arg2-arg1+arg2*arg3+arg1*arg4
GK(arg1,arg2,arg3,arg4) =
2*arg1*arg4/(GB(arg1,arg2,arg3,arg4)+sqrt(GB(arg1,arg2,arg3,arg4)^2-
4*(arg2-arg1)*arg1*arg4))

# parameters
# to simulate rapamycin treatment: mTORT = 0.3
# to simulate starvation: STARV = 0.5
p STARV=0
p kaulk=0.101, kaulk'=25, kiulk=0.075, kiulk'=20, Julk=0.001, ULK1T=1
p kaak=2, kiak=0.1, kiak'=3, kiak"=10, AMPKT=1, Jampk=0.001
p kamtor=0.0175, kimtor=0.0125, kimtor'=10, kimtor"=0.1, Jmtor=0.01,
mTORT=1
p kapr=0.01, kapr'=10, kipr=20,Jpr=0.02

done
```

The code for time course simulations

```
# a model to simulate time courses when AMPK induces ULK1 throughout an
extra protein

# initial conditions
init ULK1=0, AMPK=0.00071, ATG=0.02075

# differential equations
# ULK1 represents the active form of ULK1
ULK1' = (kaulk + kaulk'*Prot)*(Ulk1T-ULK1)/(Julk + ULK1T-ULK1) - (kiulk +
kiulk'*mTOR)*ULK1/(Julk + ULK1)

# AMPK represents the active form of AMPK
AMPK' = (kaak + STARV)*(AMPKT-AMPK)/(Jampk + AMPKT-AMPK) - (kiak +
kiak'*ULK1 + kiak"*mTOR)*AMPK/(Jampk + AMPK)

# ATG represents the active form of autophagy activator complex
ATG' = (kaau + kaau'*ULK1)*(1-ATG) - (kiau + kiau'*mTOR)*ATG

# steady state function
# mTOR represents the active form of mTORC1
mTOR = kamtor*mTORT/(kamtor + kimtor + kimtor'*AMPK + kimtor"*ULK1)
aux mTOR = mTOR

# Prot represents the active form of extra protein
Prot = GK(kapr + kapr'*AMPK,kipr,Jpr,Jpr)
aux Prot = Prot

# 'Goldbeter-Koshand' function (GK)
GB(arg1,arg2,arg3,arg4) = arg2-arg1+arg2*arg3+arg1*arg4
GK(arg1,arg2,arg3,arg4) =
2*arg1*arg4/(GB(arg1,arg2,arg3,arg4)+sqrt(GB(arg1,arg2,arg3,arg4)^2-
4*(arg2-arg1)*arg1*arg4))

# parameters
# to simulate rapamycin treatment: mTORT = 0.3
# to simulate starvation: STARV = 0.5
p STARV=0
p kaulk=0.101, kaulk'=25, kiulk=0.075, kiulk'=20, Julk=0.001, ULK1T=1
p kaak=2, kiak=0.1, kiak'=3, kiak"=10, AMPKT=1, Jampk=0.001
```

```
p kamtor=0.0175, kimtor=0.0125, kimtor'=10, kimtor"=0.1, Jmtor=0.01,
mTORT=1
p kapr=0.01, kapr'=10, kipr=20,Jpr=0.02
p kaau=0.01, kaau'=0.1, kiau=0.01, kiau'=0.1

done
```

Simulating AMPK-ULK1-mTORC1 regulatory triangle when AMPK can induce ULK1 via multi-phosphorylation

The detailed description of the elements of the theoretical models

	description
`ULK1P`	inactive mono-phosphorylated form of ULK1
`ULK1PP`	inactive di-phosphorylated form of ULK1
`ULK1PPP`	inactive tri-phosphorylated form of ULK1
`ULK1PPPP`	active fully-phosphorylated form of ULK1
`ULK1T`	total level of ULK1
`AMPK`	the active form of AMPK
`AMPKT`	total level of AMPK
`mTOR`	the active form of mTORC1
`mTORT`	total level of mTORC1
`ATG`	the active autophagy activator complex
`STARV`	level of starvation

The detailed description of the constants of the theoretical models

	description
`kaulk`	AMPK-dependent activation of ULK1
`kiulk`	mTORC1-dependent inactivation of ULK1
`alfa`	activity of ULK1A
`beta`	activity of ULK1P, ULK1PP and ULK1PPP
`kaak`	background activation of AMPK
`kiak`	background inactivation of AMPK
`kiak'`	ULK1A-dependent inactivation of AMPK
`kiak"`	mTORC1-dependent inactivation of AMPK
`Jampk`	Michaelis-constant of AMPK
`kamtor`	background activation of mTORC1
`kimtor`	background inactivation of mTORC1
`kimtor'`	AMPK-dependent inactivation of mTORC1
`kimtor"`	ULK1-dependent inactivation of mTORC1
`kaau`	background activation of autophagy
`kaau'`	ULK1A-dependent activation of autophagy
`kiau`	background inactivation of autophagy
`kiau'`	mTORC1-dependent inactivation of autophagy

The code for time course simulations

```
# a model to simulate time courses when AMPK induces ULK1 throughout multi-
step phosphorylation

# initial conditions
ULK1P=0.99945, ULK1PP=0.00055, ULK1PPP=3.035e-007, AMPK=0.00071,
ATG=0.00207

# differential equations
# ULK1P represents the inactive mono-phosphorylated form of ULK1
ULK1P' = kiulk*mTOR*ULK1PP - kaulk*AMPK*ULK1P

# ULK1PP represents the inactive di-phosphorylated form of ULK1
ULK1PP' = kaulk*AMPK*ULK1P - kiulk*mTOR*ULK1PP - kaulk*AMPK*ULK1PP +
kiulk*mTOR*ULK1PPP

# ULK1PPP represents the inactive tri-phosphorylated form of ULK1
ULKPPP' = kaulk*AMPK*ULK1PP - kiulk*mTOR*ULK1PPP - kaulk*AMPK*ULK1PPP +
kiulk*mTOR*ULK1A

# AMPK represents the active form of AMPK
AMPK' = (kaak + STARV)*(AMPKT-AMPK)/(Jampk + AMPKT-AMPK) - (kiak +
kiak'*ULK1A + kiak"*mTOR*AMPK/(Jampk + AMPK)

# ATG represents the active form of autophagy activator complex
ATG' = (kaau + kaau'*ULK1A)*(1-ATG) - (kiau + kiau'*mTOR)*ATG

# steady state function
# mTOR represents the active form of mTORC1
mTOR = kamtor*mTORT/(kamtor + kimtor + kimtor'*AMPK + kimtor"*(alfa*ULK1A +
beta*(ULKT-ULK1A)) + RAP)
aux mTOR = mTOR

# ULK1A represents the active fully-phosphorylated form of ULK1
ULK1A = ULK1T - ULK1P - ULK1PP - ULK1PPP
aux ULK1A = ULK1A

# parameters
# to simulate rapamycin treatment: mTORT = 0.3
# to simulate starvation: STARV = 0.1
# to simulate combination of mTOR inhibition and AMPK activation:
mTORT=0.3, kaak=0.4
p ULKT=1, alfa=1, beta=0.1
p STARV=0, RAP=0
p kaulk=0.75, kiulk=2
p kaak=0.1, kiak=0.001, kiak'=0.4, kiak"=0.5, AMPKT=1, Jampk=0.001
p kamtor=0.3, kimtor=0.01, kimtor'=20, kimtor"=3, Jmtor=0.01, mTORT=1
p kaau=0.01, kaau'=1, kiau=0.01, kiau'=10

done
```

II. Describing the bioinformatics analysis

Introducing the theoretical analysis of phosphorylation site search on ULK1 targets

ULK1 interactors were collected using the following online freely available databases:

- BioGrid (https://thebiogrid.org/),
- DIP (https://dip.doe-mbi.ucla.edu/dip/Main.cgi),
- MINT (https://mint.bio.uniroma2.it/),
- InnateDB (https://www.innatedb.com/),
- IntAct (https://www.ebi.ac.uk/intact/).

The first four columns of Supplementary Table 1. contain the interactors, the types of the modification, the effects of the modification and the references, respectively (Interactor, Modification, Effect and Reference).

After that, the potential specific Ser and Thr phosphorylation sites of AMPK interactors on ULK1 were identified by Group-based Prediction System 5.0 (http://gps.biocuckoo.cn/). The sequences of ULK1 interactors were downloaded from UniProt (https://www.uniprot.org/). The threshold was high under prediction. Supplementary Table 1. includes the position of Ser and Thr amino acids, the catalytic subunits of AMPK, the peptid sequence in the near of Ser and Thr amino acids and the score values. The score value was calculated by GPS algorithm to evaluate the potential of phosphorylation site. The higher the value, the more potential the residue is phosphorylated (http://gps.biocuckoo.cn/).

Then these potential phosphorylation sites were verified by NetPhos 3.1 (http://www.cbs.dtu.dk/services/NetPhos). Those phosphorylation sites were collected and checked where the phosphorylation kinase was unknown (see the NetPhos column in the Table 1.). The score above 0.500 indicates positive predictions.

The phosphorylation sites were also searched with the help of PhosphositePlus (https://www.phosphosite.org/homeAction.action). From this database, the consensus phosphorylation motif of AMPK was used, which was created with the help of several well-known phosphorylated sequences of AMPK substrates. The same amino acids within the identified potential AMPK phosphorylation sequences were marked with red colour (see the Peptid column of Table 1.).

Detailed description the legend of Supplementary Table 1.xls

Interactor	Modification	Effect	Reference	AMPK phosphorylation sites					NetPhos
				Position	Code	Enzym	Peptide	Score	Score - kinase (unknown)
the name of ULK1 interactor	the type of interaction	the effect of modification	the online scoucre of the given interaction	the position number # of S/T P'ion site	Ser (S) or The (T) P'ion site	name of the kinase	consensus P'ion sequence with red letters	evaluta the potential of P'ion site	evaluta the potential of P'ion site

Introducing the theoretical analysis of phosphorylation site search on ULK1

The potential Ser and Thr phosphorylation sites of AMPK were identified on ULK1 sequence by Group-based Prediction System 5.0 (http://gps.biocuckoo.cn/). The threshold was high under prediction. The Supplementary Table 2. includes the position of Ser and Thr amino acids, the catalytic subunits of AMPK, the peptid sequence in the near of Ser and Thr amino acids and the score values. The score value was calculated by GPS algorithm to evaluate the potential of phosphorylation. The higher the value, the more potential the residue is phosphorylated (http://gps.biocuckoo.cn/).

The phosphorylation sites were verified by NetPhos 3.1 (http://www.cbs.dtu.dk/services/NetPhos). Those phosphorylation sites were collected and checked where the phosphorylation kinase was unknown (see the NetPhos column in the Table 2.). The score above 0.500 indicates positive predictions.

The phosphorylation sites were also searched with the help of PhosphositePlus (https://www.phosphosite.org/homeAction.action). From this database, the consensus phosphorylation motif of AMPK is used, which is created with the help of several well-known phosphorylated sequences of AMPK substrates. The same amino acids within the identified potential AMPK phosphorylation sequences were marked with red colour (see the Peptid column of Supplementary Table 2.).

Phosphorylated positions are indicated with purple colour, what were already known in the literature.

Detailed description the legend of Supplementary Table 2.xls

AMPK phosphorylation sites					NetPhos
**Position**	**Code**	**Enzym**	**Peptide**	**Score**	**Score - kinase (unknown)**
the position number # of S/T P'ion site	Ser (S) or The (T) P'ion site	name of the kinase	consensus P'ion sequence with red letters	evaluta the potentia l of P'ion site	evaluta the potential of P'ion site

Consensus AMPK-dependent phosphorylation sites

PhosphoSite Plus shows the preferred Ser and Thr phosphorylation sites of AMPK kinase:

- AMPK1 (PRKAA1 gene):
  https://www.phosphosite.org/proteinAction.action?id=742&showAllSites=true

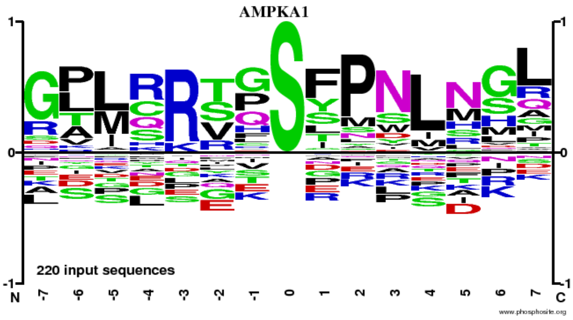

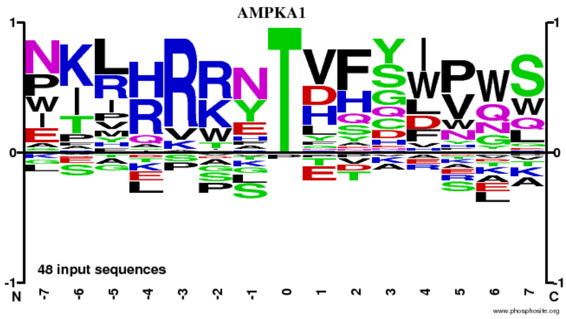

- AMPK2 (PRKAA2 gene):
  https://www.phosphosite.org/proteinAction.action?id=572&showAllSites=true

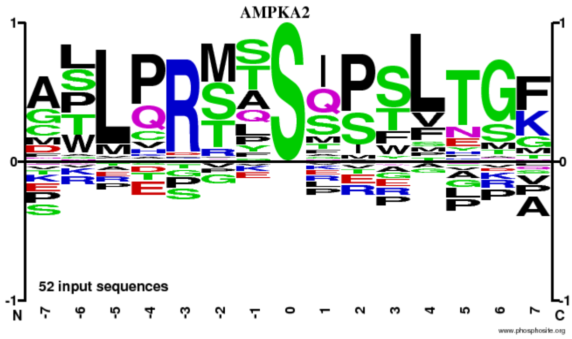

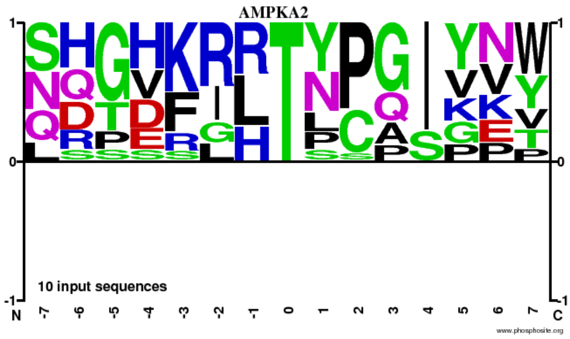

III. Supplementary Figures

Supplementary Figure 1.

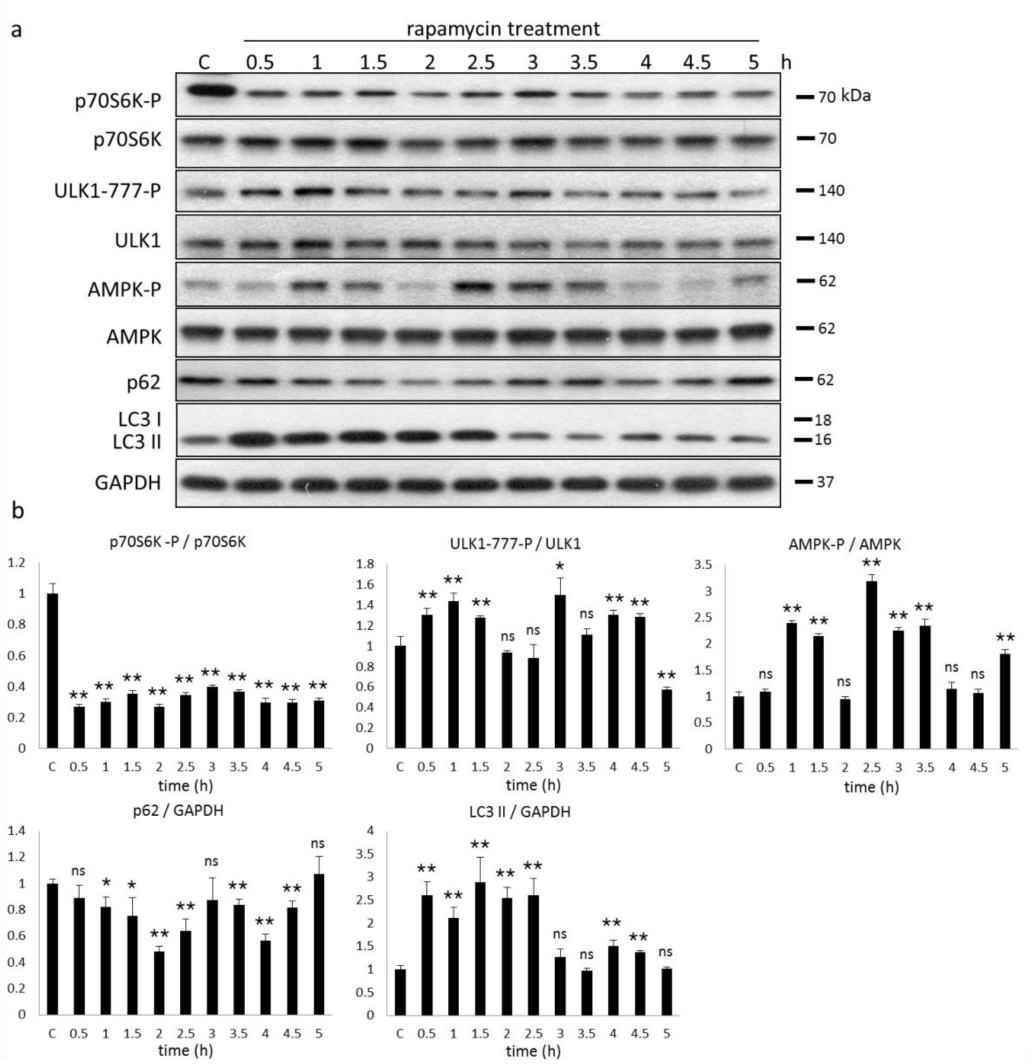

Prolonged rapamycin treatment results in oscillation of AMPK-mTORC1-ULK1 controlled autophagy in non-synchronized cell culture. (a) HEK293T cells were denoted in time after 100 nM rapamycin treatment. The markers of autophagy (p62, LC3), AMPK-P, ULK1 (ULK1-777-P) and mTORC1 (p70S6K-P) were followed by immunoblotting. GAPDH was used as loading control. **(b)** Densitometry data represent the intensity of p62 and LC3 II normalised for GAPDH, ULK1-777-P normalized for total level of ULK1, p70S6K-P normalized for total level of p70S6K and AMPK-P normalized for total level of AMPK. For each of the experiments, three independent measurements were carried out. Error bars represent standard deviation.

Supplementary Figure 2.

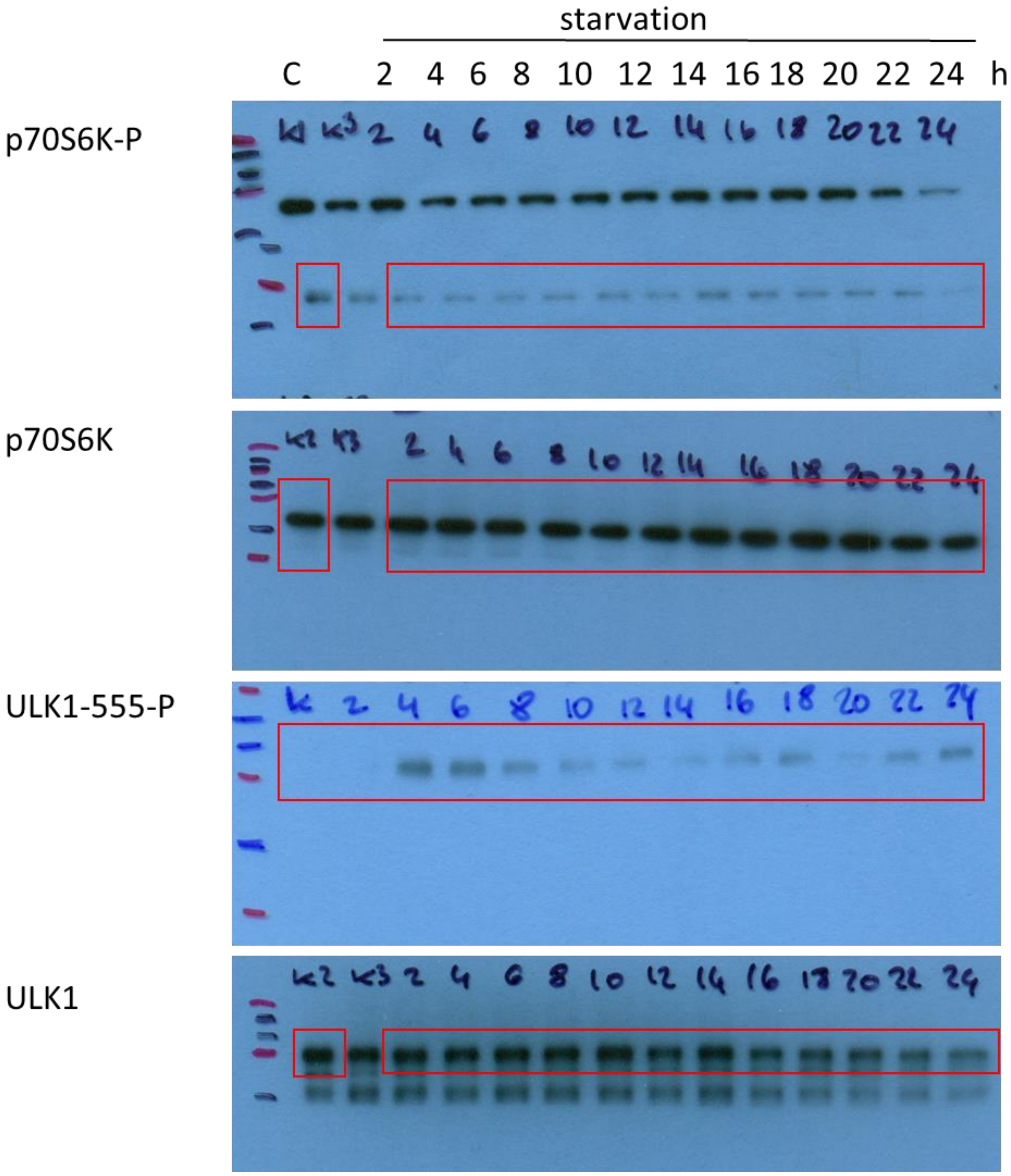

The original non-cropped version of Figure 3A. Starvation was induced in HEK293T cells by glucose depletion. The markers of ULK1 (ULK1-555-P) and mTORC1 (p70S6K-P) were followed by immunoblotting.

Supplementary Figure 3.

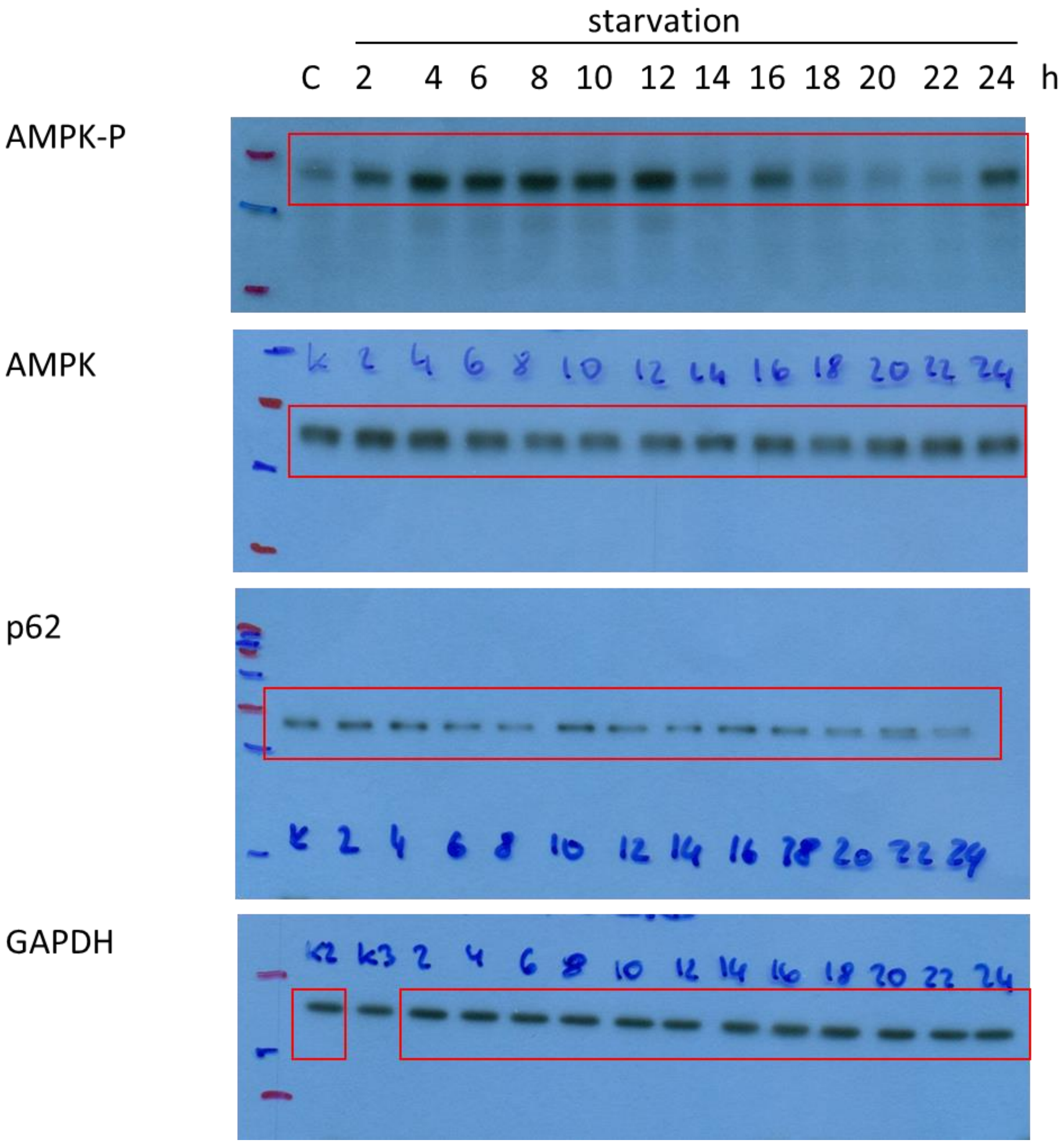

The original non-cropped version of Figure 3A. Starvation was induced in HEK293T cells by glucose depletion. The markers of AMPK-P, autophagy (p62) and GAPDH were followed by immunoblotting.

Supplementary Figure 4.

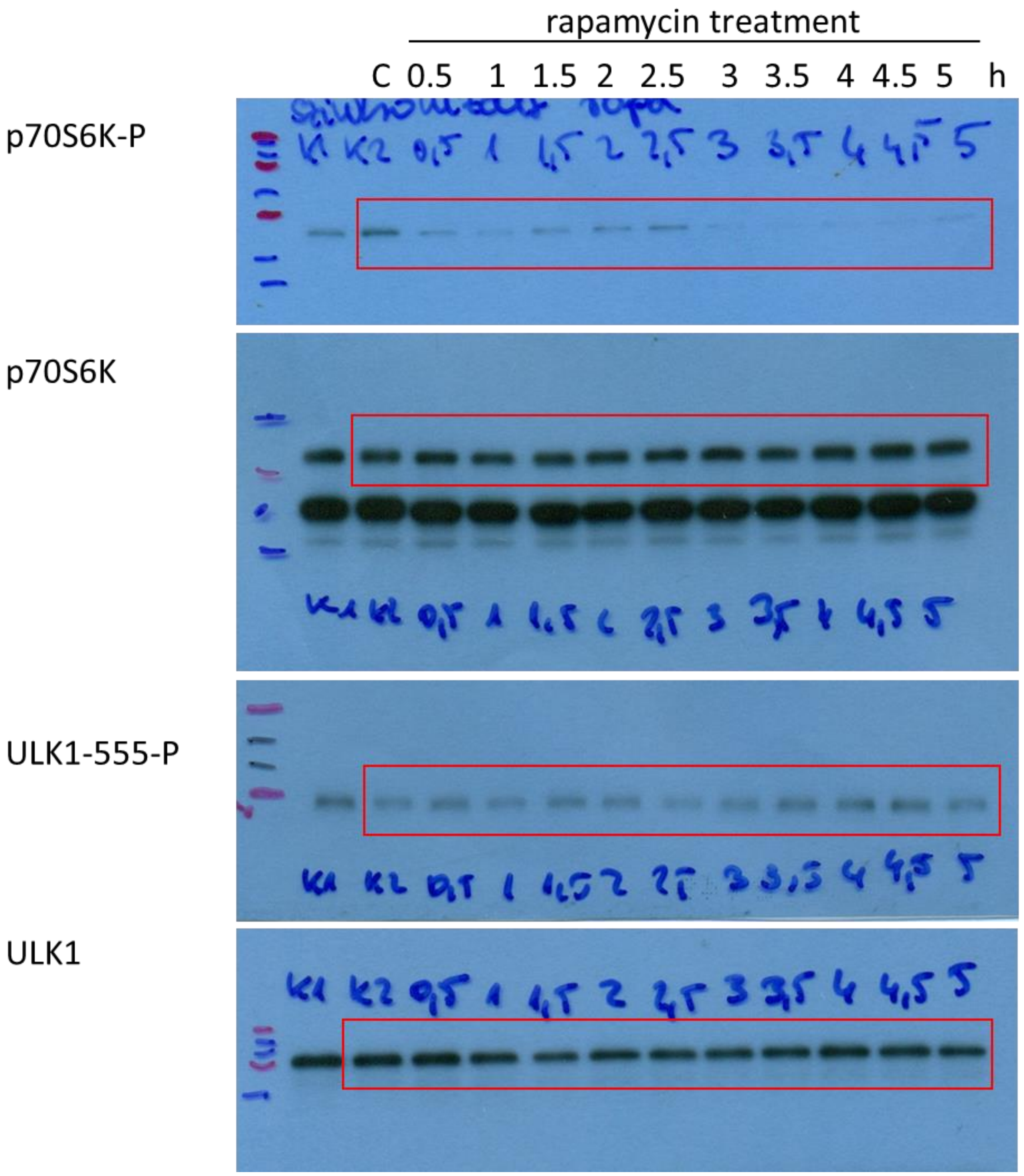

The original non-cropped version of Figure 3A. HEK293T cells were denoted in time after 100 nM rapamycin treatment. The markers of ULK1 (ULK1-555-P) and mTORC1 (p70S6K-P) were followed by immunoblotting.

Supplementary Figure 5.

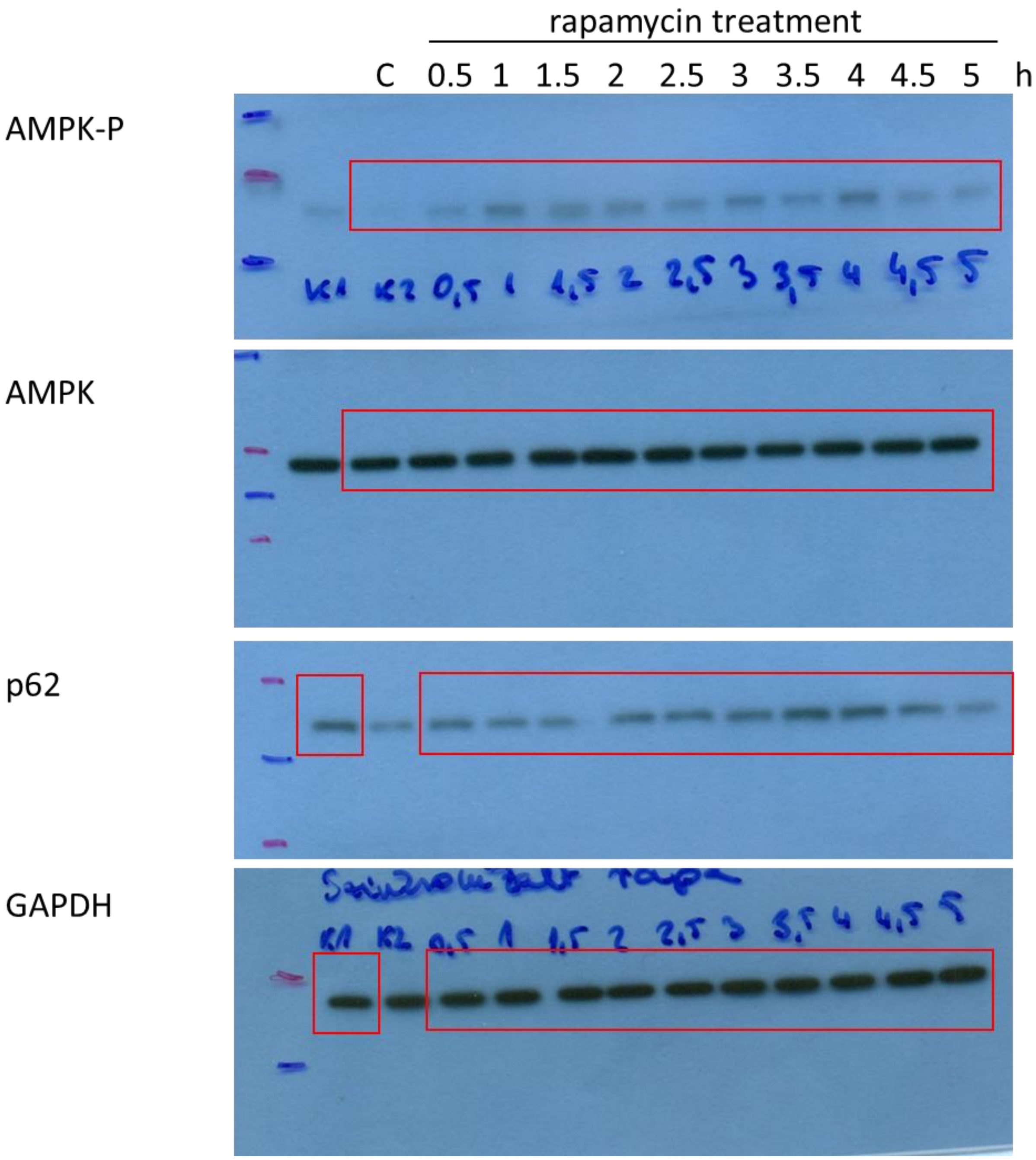

The original non-cropped version of Figure 3A. HEK293T cells were denoted in time after 100 nM rapamycin treatment. The markers of AMPK-P, autophagy (p62) and GAPDH were followed by immunoblotting.

Supplementary Figure 6.

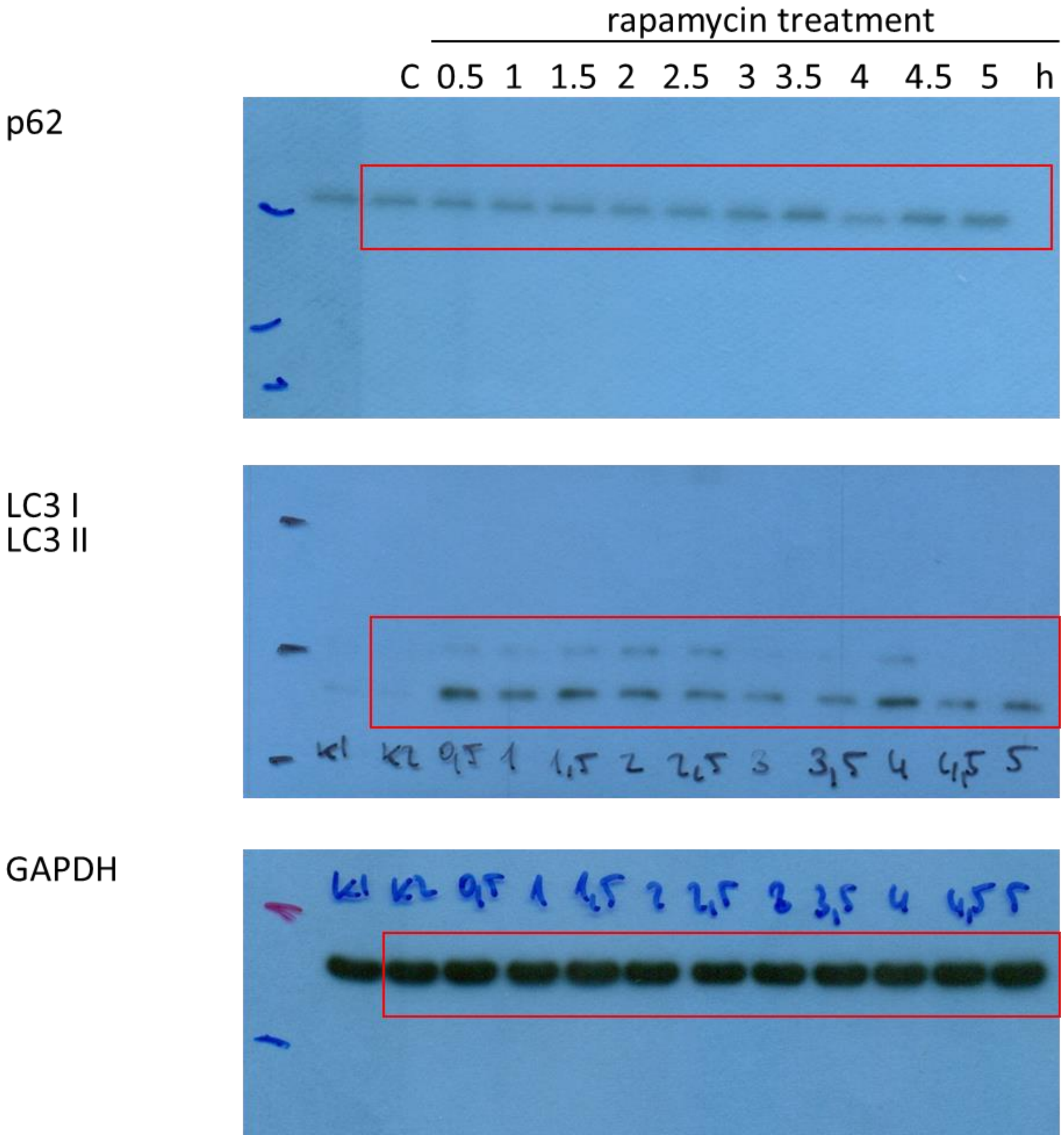

The original non-cropped version of Figure 4C. HEK293T cells were denoted in time after 100 nM rapamycin treatment. The markers of autophagy (LC3, p62) and GAPDH were followed by immunoblotting.

Supplementary Figure7.

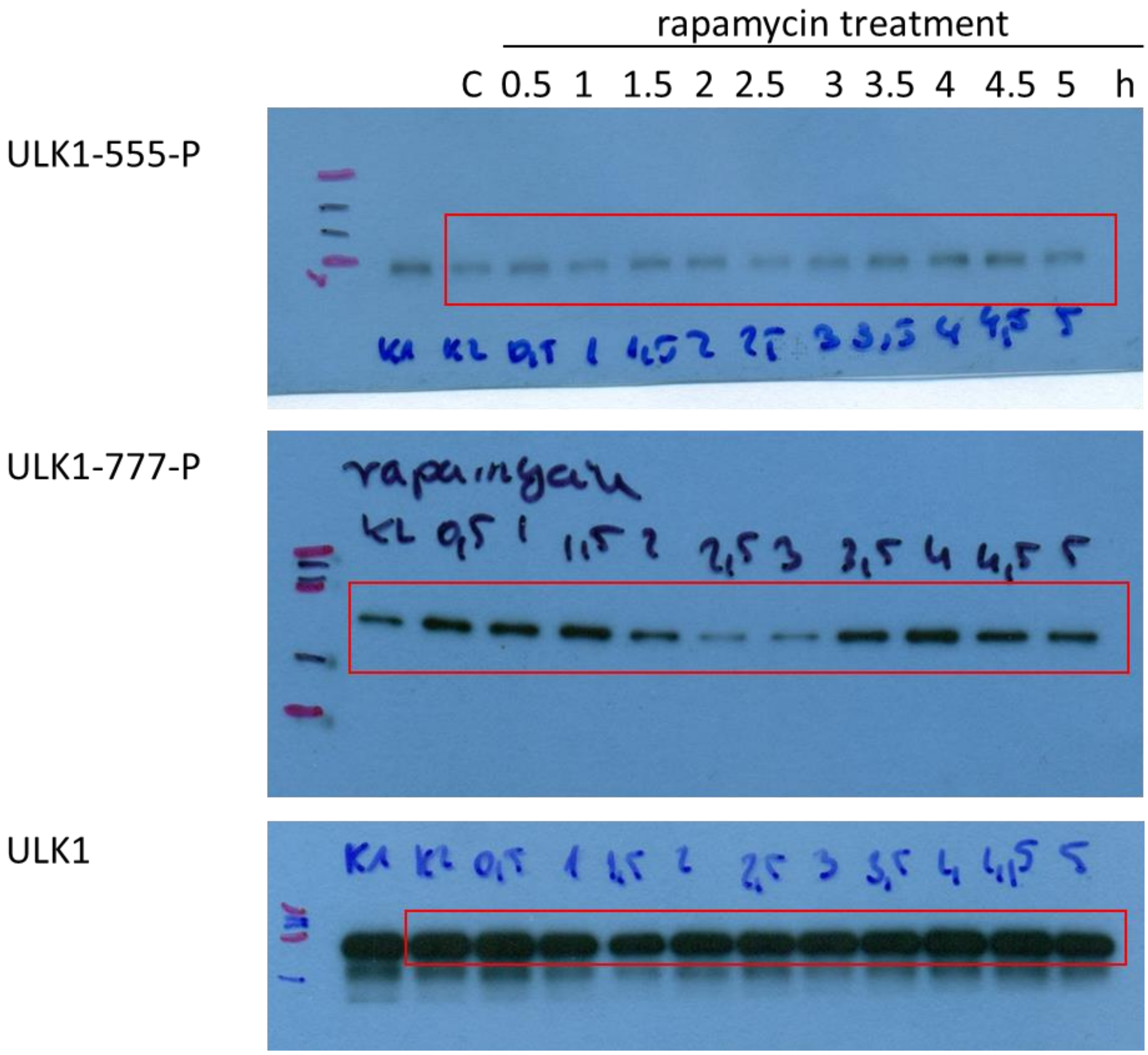

The original non-cropped version of Figure 4C. HEK293T cells were denoted in time after 100 nM rapamycin treatment. The phosphorylation of ULK1 (ULK1-777-P, ULK1-555-P) were followed by immunoblotting.

Supplementary Figure 8.

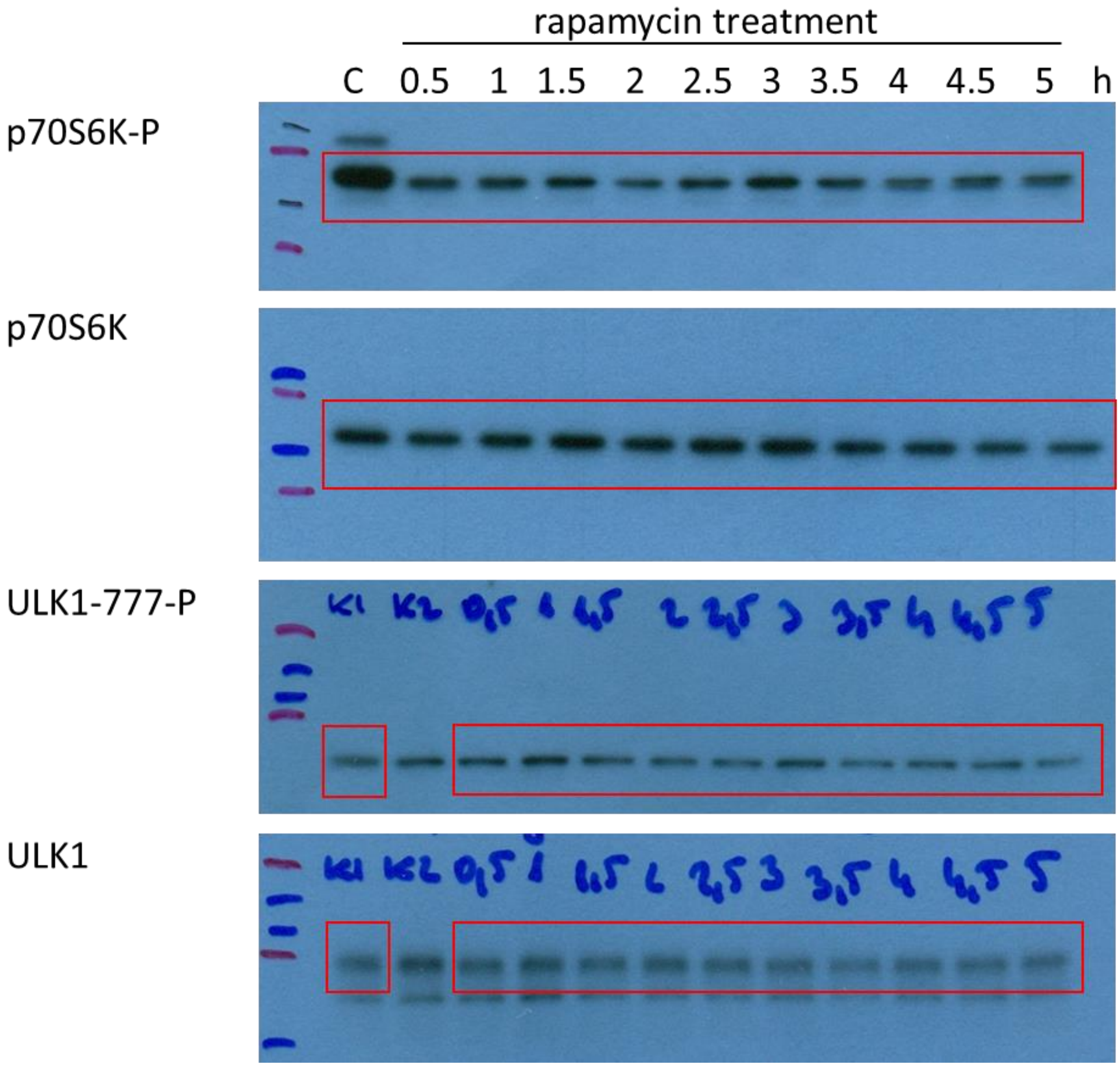

The original non-cropped version of Supplementary Figure 1. HEK293T cells were denoted in time after 100 nM rapamycin treatment without synchronisation. The markers of mTORC1 (p70S6K-P) and ULK1 (ULK1-777-P) were followed by immunoblotting.

Supplementary Figure 9.

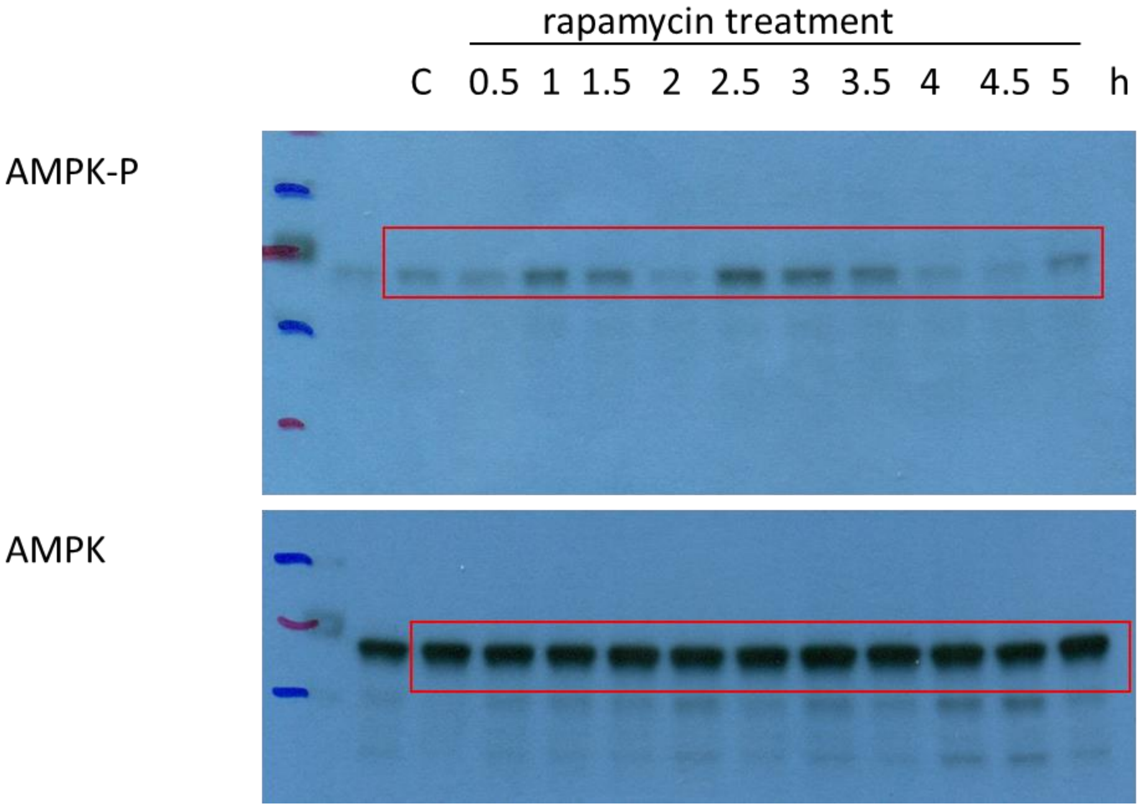

The original non-cropped version of Supplementary Figure 1. HEK293T cells were denoted in time after 100 nM rapamycin treatment without synchronisation. The markers of AMPK-P were followed by immunoblotting.

Supplementary Figure 10.

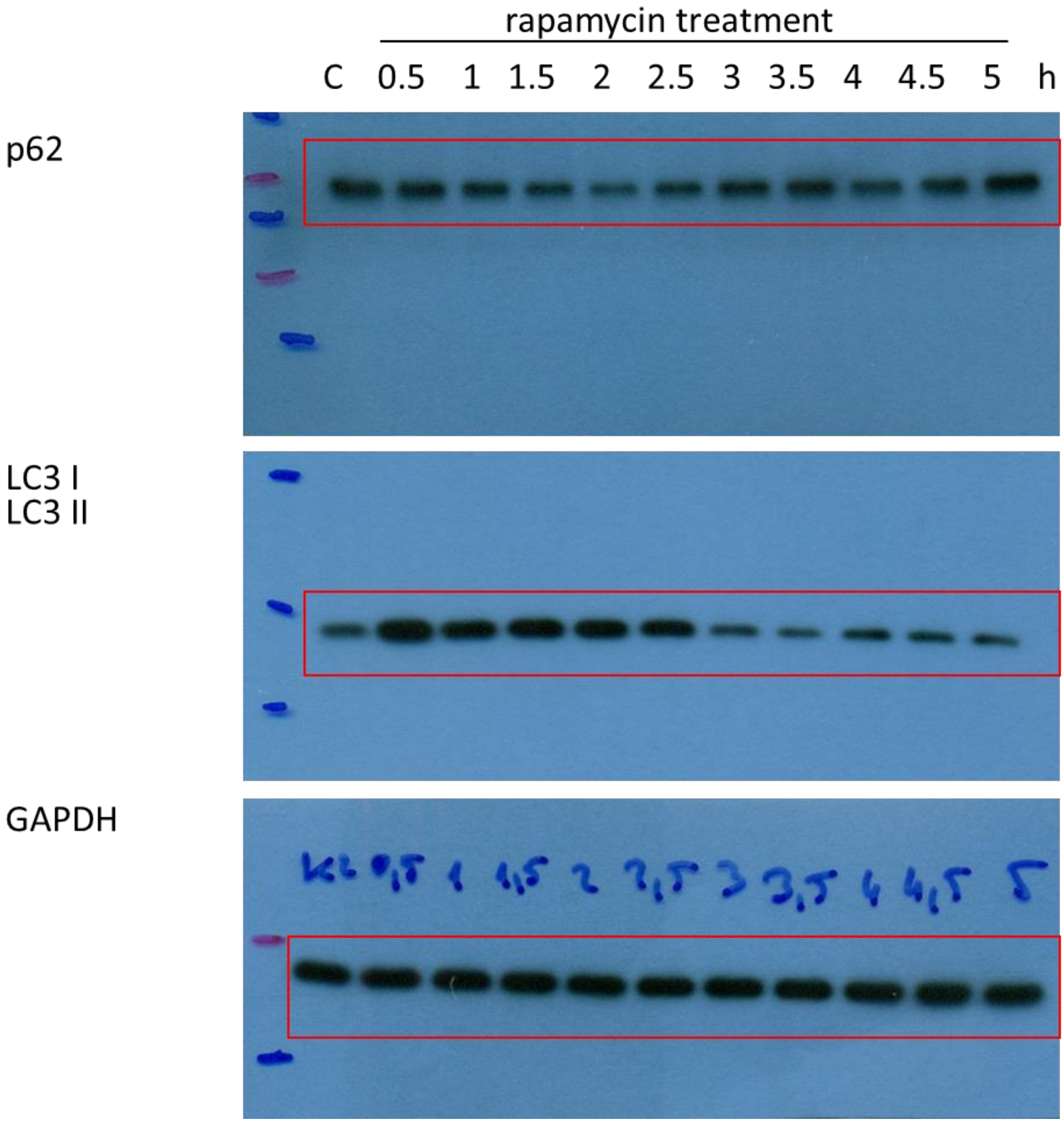

The original non-cropped version of Supplementary Figure 1. HEK293T cells were denoted in time after 100 nM rapamycin treatment without synchronisation. The markers of autophagy (LC3, p62) and GAPDH were followed by immunoblotting.

References

1. Alemu, E. A., Lamark, T., Torgersen, K. M., Birgisdottir, A. B., Larsen, K. B., Jain, A., Olsvik, H., Overvatn, A., Kirkin, V. & Johansen, T. (2012) ATG8 family proteins act as scaffolds for assembly of the ULK complex: sequence requirements for LC3-interacting region (LIR) motifs, *J Biol Chem.* **287**, 39275-90.

2. Behrends, C., Sowa, M. E., Gygi, S. P. & Harper, J. W. (2010) Network organization of the human autophagy system, *Nature.* **466**, 68-76.

3. Bodemann, B. O., Orvedahl, A., Cheng, T., Ram, R. R., Ou, Y. H., Formstecher, E., Maiti, M., Hazelett, C. C., Wauson, E. M., Balakireva, M., Camonis, J. H., Yeaman, C., Levine, B. & White, M. A. (2011) RalB and the exocyst mediate the cellular starvation response by direct activation of autophagosome assembly, *Cell.* **144**, 253-67.

4. Chauhan, S., Kumar, S., Jain, A., Ponpuak, M., Mudd, M. H., Kimura, T., Choi, S. W., Peters, R., Mandell, M., Bruun, J. A., Johansen, T. & Deretic, V. (2016) TRIMs and Galectins Globally Cooperate and TRIM16 and Galectin-3 Co-direct Autophagy in Endomembrane Damage Homeostasis, *Dev Cell.* **39**, 13-27.

5. Chauhan, S., Mandell, M. A. & Deretic, V. (2015) IRGM governs the core autophagy machinery to conduct antimicrobial defense, *Mol Cell.* **58**, 507-21.

6. Choudhury, N. R., Heikel, G., Trubitsyna, M., Kubik, P., Nowak, J. S., Webb, S., Granneman, S., Spanos, C., Rappsilber, J., Castello, A. & Michlewski, G. (2017) RNA-binding activity of TRIM25 is mediated by its PRY/SPRY domain and is required for ubiquitination, *BMC Biol.* **15**, 105.

7. Dunlop, E. A., Hunt, D. K., Acosta-Jaquez, H. A., Fingar, D. C. & Tee, A. R. (2011) ULK1 inhibits mTORC1 signaling, promotes multisite Raptor phosphorylation and hinders substrate binding, *Autophagy.* **7**, 737-47.

8. Dunlop, E. A., Seifan, S., Claessens, T., Behrends, C., Kamps, M. A., Rozycka, E., Kemp, A. J., Nookala, R. K., Blenis, J., Coull, B. J., Murray, J. T., van Steensel, M. A., Wilkinson, S. & Tee, A. R. (2014) FLCN, a novel autophagy component, interacts with GABARAP and is regulated by ULK1 phosphorylation, *Autophagy.* **10**, 1749-60.

9. Esteves, S. L., Domingues, S. C., da Cruz e Silva, O. A., Fardilha, M. & da Cruz e Silva, E. F. (2012) Protein phosphatase 1alpha interacting proteins in the human brain, *OMICS.* **16**, 3-17.

10. Hosokawa, N., Sasaki, T., Iemura, S., Natsume, T., Hara, T. & Mizushima, N. (2009) Atg101, a novel mammalian autophagy protein interacting with Atg13, *Autophagy.* **5**, 973-9.

11. Huttlin, E. L., Bruckner, R. J., Paulo, J. A., Cannon, J. R., Ting, L., Baltier, K., Colby, G., Gebreab, F., Gygi, M. P., Parzen, H., Szpyt, J., Tam, S., Zarraga, G., Pontano-Vaites, L., Swarup, S., White, A. E., Schweppe, D. K., Rad, R., Erickson, B. K., Obar, R. A., Guruharsha, K. G., Li, K., Artavanis-Tsakonas, S., Gygi, S. P. & Harper, J. W. (2017) Architecture of the human interactome defines protein communities and disease networks, *Nature.* **545**, 505-509.

12. Joachim, J., Razi, M., Judith, D., Wirth, M., Calamita, E., Encheva, V., Dynlacht, B. D., Snijders, A. P., O'Reilly, N., Jefferies, H. B. J. & Tooze, S. A. (2017) Centriolar Satellites Control GABARAP Ubiquitination and GABARAP-Mediated Autophagy, *Curr Biol.* **27**, 2123-2136 e7.

13. Jung, C. H., Jun, C. B., Ro, S. H., Kim, Y. M., Otto, N. M., Cao, J., Kundu, M. & Kim, D. H. (2009) ULK-Atg13-FIP200 complexes mediate mTOR signaling to the autophagy machinery, *Mol Biol Cell.* **20**, 1992-2003.

14. Kimura, T., Jain, A., Choi, S. W., Mandell, M. A., Schroder, K., Johansen, T. & Deretic, V. (2015) TRIM-mediated precision autophagy targets cytoplasmic regulators of innate immunity, *J Cell Biol.* **210**, 973-89.

15. Kumar, A. & Shaha, C. (2018) SESN2 facilitates mitophagy by helping Parkin translocation through ULK1 mediated Beclin1 phosphorylation, *Sci Rep.* **8**, 615.

16. Li, J., Chen, Z., Stang, M. T. & Gao, W. (2017) Transiently expressed ATG16L1 inhibits autophagosome biogenesis and aberrantly targets RAB11-positive recycling endosomes, *Autophagy.* **13**, 345-358.

17. Li, J., Qi, W., Chen, G., Feng, D., Liu, J., Ma, B., Zhou, C., Mu, C., Zhang, W., Chen, Q. & Zhu, Y. (2015) Mitochondrial outer-membrane E3 ligase MUL1 ubiquitinates ULK1 and regulates selenite-induced mitophagy, *Autophagy*. **11**, 1216-29.

18. Longatti, A., Lamb, C. A., Razi, M., Yoshimura, S., Barr, F. A. & Tooze, S. A. (2012) TBC1D14 regulates autophagosome formation via Rab11- and ULK1-positive recycling endosomes, *J Cell Biol*. **197**, 659-75.

19. McKenzie, J. A., Riento, K. & Ridley, A. J. (2006) Casein kinase I epsilon associates with and phosphorylates the tight junction protein occludin, *FEBS Lett*. **580**, 2388-94.

20. McKnight, N. C., Jefferies, H. B., Alemu, E. A., Saunders, R. E., Howell, M., Johansen, T. & Tooze, S. A. (2012) Genome-wide siRNA screen reveals amino acid starvation-induced autophagy requires SCOC and WAC, *EMBO J*. **31**, 1931-46.

21. Mercer, C. A., Kaliappan, A. & Dennis, P. B. (2009) A novel, human Atg13 binding protein, Atg101, interacts with ULK1 and is essential for macroautophagy, *Autophagy*. **5**, 649-62.

22. Mukhopadhyay, S., Naik, P. P., Panda, P. K., Sinha, N., Das, D. N. & Bhutia, S. K. (2016) Serum starvation induces anti-apoptotic cIAP1 to promote mitophagy through ubiquitination, *Biochem Biophys Res Commun*. **479**, 940-946.

23. Nazio, F., Carinci, M., Valacca, C., Bielli, P., Strappazzon, F., Antonioli, M., Ciccosanti, F., Rodolfo, C., Campello, S., Fimia, G. M., Sette, C., Bonaldo, P. & Cecconi, F. (2016) Fine-tuning of ULK1 mRNA and protein levels is required for autophagy oscillation, *J Cell Biol*. **215**, 841-856.

24. Nazio, F., Strappazzon, F., Antonioli, M., Bielli, P., Cianfanelli, V., Bordi, M., Gretzmeier, C., Dengjel, J., Piacentini, M., Fimia, G. M. & Cecconi, F. (2013) mTOR inhibits autophagy by controlling ULK1 ubiquitylation, self-association and function through AMBRA1 and TRAF6, *Nat Cell Biol*. **15**, 406-16.

25. Okazaki, N., Yan, J., Yuasa, S., Ueno, T., Kominami, E., Masuho, Y., Koga, H. & Muramatsu, M. (2000) Interaction of the Unc-51-like kinase and microtubule-associated protein light chain 3 related proteins in the brain: possible role of vesicular transport in axonal elongation, *Brain Res Mol Brain Res*. **85**, 1-12.

26. Park, J. M., Jung, C. H., Seo, M., Otto, N. M., Grunwald, D., Kim, K. H., Moriarity, B., Kim, Y. M., Starker, C., Nho, R. S., Voytas, D. & Kim, D. H. (2016) The ULK1 complex mediates MTORC1 signaling to the autophagy initiation machinery via binding and phosphorylating ATG14, *Autophagy*. **12**, 547-64.

27. Pei, G., Buijze, H., Liu, H., Moura-Alves, P., Goosmann, C., Brinkmann, V., Kawabe, H., Dorhoi, A. & Kaufmann, S. H. E. (2017) The E3 ubiquitin ligase NEDD4 enhances killing of membrane-perturbing intracellular bacteria by promoting autophagy, *Autophagy*. **13**, 2041-2055.

28. Polager, S., Ofir, M. & Ginsberg, D. (2008) E2F1 regulates autophagy and the transcription of autophagy genes, *Oncogene*. **27**, 4860-4.

29. Popovic, D. & Dikic, I. (2014) TBC1D5 and the AP2 complex regulate ATG9 trafficking and initiation of autophagy, *EMBO Rep*. **15**, 392-401.

30. Pyo, K. E., Kim, C. R., Lee, M., Kim, J. S., Kim, K. I. & Baek, S. H. (2018) ULK1 O-GlcNAcylation Is Crucial for Activating VPS34 via ATG14L during Autophagy Initiation, *Cell Rep*. **25**, 2878-2890 e4.

31. Qin, L., Tian, Y., Yu, Z., Shi, D., Wang, J., Zhang, C., Peng, R., Chen, X., Liu, C., Chen, Y., Huang, W. & Deng, W. (2016) Targeting PDK1 with dichloroacetophenone to inhibit acute myeloid leukemia (AML) cell growth, *Oncotarget*. **7**, 1395-407.

32. Ro, S. H., Semple, I. A., Park, H., Park, H., Park, H. W., Kim, M., Kim, J. S. & Lee, J. H. (2014) Sestrin2 promotes Unc-51-like kinase 1 mediated phosphorylation of p62/sequestosome-1, *FEBS J*. **281**, 3816-27.

33. Rui, Y. N., Xu, Z., Patel, B., Chen, Z., Chen, D., Tito, A., David, G., Sun, Y., Stimming, E. F., Bellen, H. J., Cuervo, A. M. & Zhang, S. (2015) Huntingtin functions as a scaffold for selective macroautophagy, *Nat Cell Biol*. **17**, 262-75.

34. Sugis, E., Dauvillier, J., Leontjeva, A., Adler, P., Hindie, V., Moncion, T., Collura, V., Daudin, R., Loe-Mie, Y., Herault, Y., Lambert, J. C., Hermjakob, H., Pupko, T., Rain, J. C., Xenarios, I., Vilo, J., Simonneau, M. & Peterson, H. (2019) HENA, heterogeneous network-based data set for Alzheimer's disease, *Sci Data.* **6**, 151.

35. Tomoda, T., Kim, J. H., Zhan, C. & Hatten, M. E. (2004) Role of Unc51.1 and its binding partners in CNS axon outgrowth, *Genes Dev.* **18**, 541-58.

36. Webster, C. P., Smith, E. F., Bauer, C. S., Moller, A., Hautbergue, G. M., Ferraiuolo, L., Myszczynska, M. A., Higginbottom, A., Walsh, M. J., Whitworth, A. J., Kaspar, B. K., Meyer, K., Shaw, P. J., Grierson, A. J. & De Vos, K. J. (2016) The C9orf72 protein interacts with Rab1a and the ULK1 complex to regulate initiation of autophagy, *EMBO J.* **35**, 1656-76.

37. Wong, P. M., Feng, Y., Wang, J., Shi, R. & Jiang, X. (2015) Regulation of autophagy by coordinated action of mTORC1 and protein phosphatase 2A, *Nat Commun.* **6**, 8048.

38. Zhang, Y., Burberry, A., Wang, J. Y., Sandoe, J., Ghosh, S., Udeshi, N. D., Svinkina, T., Mordes, D. A., Mok, J., Charlton, M., Li, Q. Z., Carr, S. A. & Eggan, K. (2018) The C9orf72-interacting protein Smcr8 is a negative regulator of autoimmunity and lysosomal exocytosis, *Genes Dev.* **32**, 929-943.
